# Evolution of Shore Hardness under Uniaxial Tension/Compression in Body-Temperature Programmable Elastic Shape Memory Hybrids

**DOI:** 10.3390/polym14224872

**Published:** 2022-11-11

**Authors:** Balasundaram Selvan Naveen, Nivya Theresa Jose, Pranav Krishnan, Subham Mohapatra, Vivek Pendharkar, Nicholas Yuan Han Koh, Woon Yong Lim, Wei Min Huang

**Affiliations:** 1 School of Mechanical and Aerospace Engineering, Nanyang Technological University, 50 Nanyang Avenue, Singapore 639798, Singapore; 2 Polymer Science and Engineering, Indian Institute of Technology, Roorkee 247667, India; 3 Department of Metallurgical and Materials Engineering, Indian Institute of Technology, Kharagpur 721302, India; 4 Department of Mechanical Engineering, National Institute of Technology, Rourkela 769008, India; 5 Department of Metallurgical Engineering and Materials Science, Indian Institute of Technology Bombay, Mumbai 400076, India

**Keywords:** shore hardness, shape memory effect, shape memory hybrids, debonding, micro-cracks, cyclic loading, programming, recovery, Mullins effect

## Abstract

Body-temperature programmable elastic shape memory hybrids (SMHs) have great potential for the comfortable fitting of wearable devices. Traditionally, shore hardness is commonly used in the characterization of elastic materials. In this paper, the evolution of shore hardness in body-temperature programmable elastic SMHs upon cyclic loading, and during the shape memory cycle, is systematically investigated. Upon cyclic loading, similar to the Mullins effect, significant softening appears, when the applied strain is over a certain value. On the other hand, after programming, in general, the measured hardness increases with increase in programming strain. However, for certain surfaces, the hardness decreases slightly and then increases rapidly. The underlying mechanism for this phenomenon is explained by the formation of micro-gaps between the inclusion and the matrix after programming. After heating, to melt the inclusions, all samples (both cyclically loaded and programmed) largely recover their original hardness.

## 1. Introduction

Shape memory materials (SMMs) are able to recover their original shape, but only when a particular stimulus is applied [1,2,3]. The underlying mechanisms for different types of SMMs are different [4]. The idea of shape memory hybrids (SMHs) provides a convenient approach for almost anyone to design a special SMM with tailored thermo-mechanical/shape memory properties to meet all the requirements of a particular application [5].

Basically, an SMH includes two parts: one is the elastic part, which is always highly elastic during the whole application process, and the other part is the transition part, which controls the shape memory features of the SMH [5]. Mostly, we select an elastic polymeric or metallic material for the elastic part, while the selection of the transition part is determined by the requirements of the application, so that the transition part might be organic, non-organic (including water), or metal (including alloys) [3,6].

Body-temperature programmable SMMs feature the capability to be reshaped into a temporary shape at around the body temperature [7,8]. Hence, they are highly in demand for the comfortable fitting of wearable devices [9,10]. A typical example is polycaprolactone (PCL), which melts at about 70 °C upon heating and, then, crystallizes at around body temperature in a few minutes [11,12,13]. Thus, differing from glass transition-based body-temperature programmable SMMs (e.g., [8,14]), not only is there enough time to fit PCL-based shape memory splints to provide strong support, but also there is no need to worry that the splints may become softer when the body temperature increases due to a fever or infection, or when the ambient temperature rises.

Following the concept of SMH, we developed highly elastic SMMs, in which soft silicone is used as the elastic part [15]. If PCL, or a similar material, is used for the transition part, the additional feature of body-temperature programmability is achieved as well [9,10]. Such materials, in foam shapes, are ideal for cushions and insoles to replace ethylene vinyl acetate (EVA) or polyurethane (PU) [14]. However, softening occurs in these materials when they undergo deformation of any kind, similar to what Mullins observed in vulcanizates either with or without reinforcing fillers [16]. This can be explicitly seen in a stress vs. strain curve determined during the first loading/unloading cycle. Subsequent repeated cycles of the same deformation cause the material to approach a steady state with a constant stress vs. strain curve. For SMHs, the influence of programming and recovery in a shape memory cycle on the hardness needs to be further investigated.

The evolution of the hardness of some elastic materials is directly linked to the presence of the Mullins effect, which has been studied for over seven decades, but still physical understanding of this behavior has yet to be well understood [17]. The Mullins effect is observed not only in tension, but also in other types of loading, such as compression [18,19], shearing [20,21], hydrostatic tension [22] and equi-biaxial tension [23,24,25,26]. The Mullins effect is more pronounced in natural rubber reinforced with filler materials for reinforcement [27,28,29]. The presence of the Mullins effect in the form of stress softening in carbon black reinforced rubber was reported in [30], and, in commercial silicone elastomers, in [31,32,33]. Mullins also noted that the higher the residual strain after unloading, the more significant the softening effect [34,35,36,37]. For elastomers with embedded filler particles, the main reason for the Mullins effect is the breakage of bonds between the polymer chains and the filler particles [38]. Molecular slippage was also proposed as a mechanism for the Mullins effect in elastomers filled with carbon black [39]. The effect of filler content in a rubber on the Mullins effect is studied extensively by [28] which reported that the main mechanism of the Mullins effect, especially with low filler content, was due to the rubber molecular chain shedding and slippage from the surface of the fillers. At higher filler content, the breakage of the filler–filler 3D network during stretching is reported to be the main reason for the Mullins effect in the rubber blend. An interesting observation was made in [31], which reported that the Mullins effect could be reduced by applying hydrophobic surface treatment on silica filled elastomers. Such a surface treatment greatly reduced chain entanglement at particle morphology, which then led to reduction in the formation of silica agglomerates and, in turn, reduced the Mullins effect during stretching as lower agglomerates resulted in lower breakage of silica–silica fillers, thereby leading to a lowering of the Mullins effect.

The Mullins effect can be removed to a great extent [17]. It was claimed in [40] that a rest period of four weeks at room temperature resulted in partial stress recovery in a natural rubber filled with carbon black. The recovery of the Mullins effect upon heat treatment of rubber materials was reported in [41,42,43]. For instance, in [16], 80% recovery was achieved when heated at 100 °C for two days. The temperature-dependent nature of the Mullins effect in thermoplastic vulcanizates was reported in [28,44] and it was reported that it could be enhanced by increasing the heat treatment temperature. High temperature makes it possible for the broken polymeric chains to regenerate, thereby reversing the softening of the material due to the Mullins effect [45]. As mentioned in [46], the formation of new networks and crosslinks in the rubber at high temperatures could be the cause of the recovery. Instead of heating, in [47], a natural rubber was immersed in a suitable solvent to get rid of the Mullins effect through swelling. Although the presence of the Mullins effect upon cyclic loading in elastic SMHs is well documented [9,10,15], so far there is no report about how to eliminate this effect.

Shore hardness, measured by Durometer, is one of the most frequently used tests to measure the hardness of highly elastic materials [48]. The simple and non-destructive nature of this test makes it suitable for the quick testing of highly elastic materials [49]. Hardness values can be linked directly to the Young’s modulus of a material [50]. The harder the material, the higher its Young’s modulus. The influence of inclusions on the hardness of the overall matrix was explored in [51]. Since the inclusions are typically much harder than the elastic matrix, with an increase in the quantity of inclusions, the hardness of the overall sample increases.

In this study, body-temperature programmable elastic shape memory hybrids were produced using elastic silicone as the elastic part and PCL as the transition part. In addition to stress vs. strain responsiveness in both cyclic tension and cyclic compression, their shape memory performance, in terms of the shape fixity ratio and shape recovery ratio, were also characterized. The evolution of shore hardness of these samples was monitored to investigate the effects of cyclic tension/compression on different strains, programming to different strains and recovery after heating of samples after cyclic loading and after programming.

Finite element simulation was also carried out to reveal the influence of the distribution of the inclusions on the stress vs. strain response in loading, and the effects of bonding between the elastic matrix and inclusions. Additional experiments were conducted to demonstrate the formation of micro gaps between the inclusion and matrix after programming.

## 2. Materials, Samples Preparation and Experimental

### 2.1. Materials

The materials used in all SMH samples (cylindrical, cuboid and strip) were M10 silicone (with a shore hardness of 10A, from Lianhuan Group Limited, Shenzhen, China) as the elastic part and PCL (in pellet form, molecular weight: 60,000, from Shenzhen Esun Industrial Co., Ltd., Shenzhen, China) as the transition part.

The silicone had good elasticity and had a wide operating temperature in the range of −50 °C to 200 °C. The silicone came in two parts, part A was the silicone monomer and part B the curing agent. Both parts came in liquid form. It was recommended b the manufacturer to mix the parts in 40:1 weight ratio of A:B, with a curing time of around ten minutes at room temperature. However, ten minutes was too short to remove the air bubbles formed during the mixing of part A and part B. Hence, we selected a mixing ratio of 55:1 which took about two hours to cure at room temperature (about 25 °C). This gave us ample time to get rid of the air bubbles before the sample was cured. The density of the cured silicone was 1.03 g/cm^3^ at room temperature.

PCL was chosen as the transition part for this study. The melting temperature of the PCL was around 60–70 °C [52], and the density was 1.1 g/cm^3^. As reported in [9,10], PCL can be crystallized at body temperature of around 37 °C. This property is useful for comfort fitting as the user can program the shape to suit his/her needs at body temperature, instead of at higher temperatures, where it might be too hot to handle comfortably, as reported in [10].

### 2.2. Sample Preparation

All the samples were produced with a silicone to PCL weight ratio of 56:40 in three different shapes for this study. The ratio of 56:40 was selected because this ratio provided the best shape fixity ratios for the produced samples. According to [5], the lower the amount of PCL in an SMH, the lesser its shape fixity ratio. However, with more than 40% (volume ratio) of PCL, PCL inclusions turn to aggregates within the elastic matrix (silicone) and, consequently, the resulting SMHs become non-uniform and inconsistent.

The samples were prepared according to the following process. Initially, 55 g of part A silicone monomer and 40 g of PCL pellets were added together in a beaker and then heated at 90 °C for 15 min in an oven. Then, the beaker was retrieved, and the mixture stirred vigorously for five minutes to make sure that the PCL was dispersed uniformly in the silicone. Subsequently, the mixture was cooled to room temperature. In the next step, 1 g of part B curing agent was added into the mixture and the mixture was stirred for five minutes to ensure that the curing agent mixed thoroughly. After this, the mixture was poured into appropriate molds and then left to cure at room temperature. The samples cured in around two hours. Finally, the samples were retrieved from the molds for testing. Table 1 presents the dimensions of the samples prepared and the corresponding tests performed on them in this study.

The procedure to prepare the SMH samples was essentially the same as that reported in, for instance, [5,9,10,15]. Hence, the distribution of PCL inclusions and their shape evolution (including the eye-ball phenomenon, due to debonding between the spherical inclusions and matrix [15]) during mechanical and shape memory cycling should be about the same.

### 2.3. Experimental Procedure

Unless otherwise stated, the stress and strain in this study refer to engineering stress and engineering strain, respectively. All the cyclic compression tests, cyclic tensile tests and programming compressions were carried out using a Shimadzu AG-10kNXplus STD (Kyoto, Japan) universal testing machine.

A complete shape memory cycle, including programming and recovery processes, reveals the shape memory performance of a polymeric shape memory material [53]. Soft silicones are highly viscoelastic. Thus, not only is the influence of strain rate significant [9,10,15], but also creeping and relaxation are apparent [54]. In this study, we only considered the corresponding performance when the material was relatively stable.

The typical testing procedure (for cylindrical samples) was as follows. The basic procedure for conducting the shape memory test was the same as that reported in [10].

(1) An SMH cylindrical sample was heated to 80 °C, which is above the melting temperature of PCL [10], for around 15 min in an oven.

(2) The sample was retrieved from the oven and then cooled down to 37 °C (body temperature).

(3) The sample was then compressed to a prescribed programming compressive strain ($\varepsilon_{m})$ in the above-mentioned universal testing machine at a strain rate of 10^−1^/s. A heating chamber was used concurrently with the universal testing machine to maintain a temperature of 37 °C on the sample throughout the programming process.

(4) Subsequently, the heating chamber was removed and the sample was allowed to recrystallize upon cooling down to room temperature, while maintaining the compressive strain for 30 min.

(5) Unloading was carried out and the programmed sample was retrieved from the machine.

This completed the programming process of the shape memory cycle [53]. The residual strain ($\varepsilon_{1})$ in the sample was noted.

Finally, the sample was heated to 80 °C for 15 min. The remaining strain ($\varepsilon_{2})$ was noted, and this marked the end of the recovery process [53].

Shape fixity ratio (*R_f_*) and recovery ratio (*R_r_*) of the sample were calculated by [5,53]:

(1) $$R_{f}=\frac{\varepsilon_{1}}{\varepsilon_{m}}$$

(2) $$R_{r}=\frac{\varepsilon_{1}-\varepsilon_{2}}{\varepsilon_{1}}$$

In this study, seven different programming compressive strains, of 10%, 20%, 30%, 40%, 50%, 60% and 70%, were used to analyze the shape memory performance of the cylindrical and cuboid samples for compression. Similar steps were followed for analyzing the shape memory performance of the strip samples programmed via stretching.

Uniaxial cyclic compression tests were performed on the cylindrical and cuboid samples and identical cyclic tensile tests were performed on the strip samples. All the cyclic tests were done at 10, 20, 30, 40, 50, 60 and 70% strains on all the samples at a constant strain rate of 10^−3^/s. This test followed the same methodology as reported in [10].

For each type of test, two different pieces were tested for their shape memory performance. For each piece, the shape fixity tests were repeated three times for each programming strain.

Shore hardness (A) of the samples was measured using a SanLiang Shore hardness tester 324-211 LX-A (Dongguan, China). They were measured for the original samples, after programming the samples to different programming compressive strains of 10%, 20%, 30%, 40%, 50%, 60% and 70%, respectively, and after heating for recovery. Shore hardness was also recorded after performing cyclic compression tests on the same samples at different programming strains of 10%, 20%, 30%, 40%, 50%, 60% and 70%, respectively, in an incremental manner. At each programming strain, five cycles were performed on these samples. At the end of each programming strain, the samples were unloaded, and their shore hardness values were measured. Then, the samples were heated for recovery. Finally, the shore hardness was again measured after the samples hardened.

Each type of hardness measurement was repeated five times at different locations spaced at 2 mm from the preceding measurement location on the same surface. The mean value of these five measurements was considered to be the actual hardness value of the sample for that particular test.

The same procedure (as used for cylindrical samples) was followed for cuboid samples. Shore hardness values were noted on the four faces of the samples (top, bottom, front and left) with the compressions performed on the top side.

A similar procedure was also applied for the strip samples, with the only difference being that all the tests were performed in tension, rather than in compression. The same programming strains were used and shore hardness values were measured in the same manner on the top surface (thickness direction) of the strip samples that were subjected to tension along the length direction. Here, two samples (Sample I and Sample II) prepared for the test were slightly different. Sample I was hardened with slightly more hardener. Consequently, Sample I was a bit harder than other samples.

## 3. Results and Analysis

**Figure 1:**
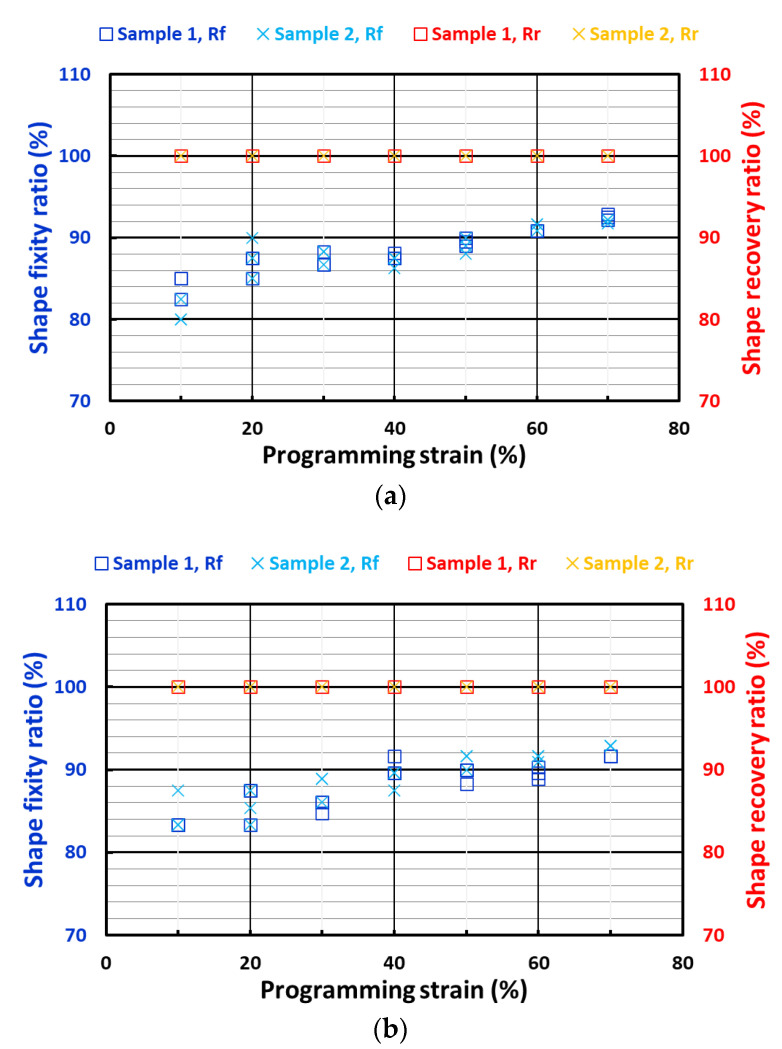

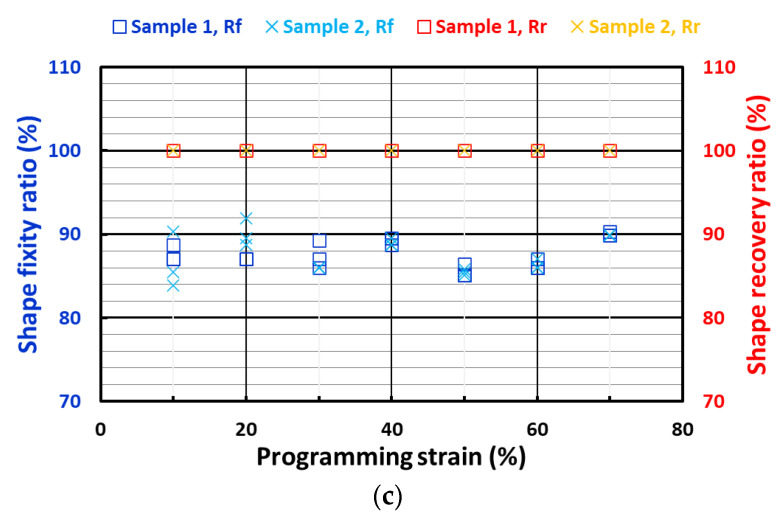
Shape fixity ratios and shape recovery ratios of (**a**) cylindrical samples; (**b**) cuboid samples; and (**c**) strip samples.

Figure 1 shows the shape fixity ratios and shape recovery ratios of all the three types of samples (cylindrical, cuboid and strip) investigated in this study. It can be seen that the shape recovery ratios for all the samples were 100%, since the silicone used in this study is highly elastic in the operating range of these tests [5].

In general, the shape fixity ratios of all samples were around 81 to 91%. The shape fixity ratios of the cylindrical and cuboid samples (which were subjected to compression programming) increased slightly as the programming strain increased. However, this was not the case for the strip samples (programmed via stretching) which had similar shape fixity ratios at all programming strains. According to [55], a higher programming strain results in a higher shape fixity ratio, while the programming temperature is the other influential parameter. Thus, the shape fixity ratio of a sample was also affected by the possible temperature non-uniformity in the tested samples. As mentioned above, the programming of theses samples started when the surface temperature reached 37 °C. The temperature gradient in the thin strip samples tended be less significant than those in the cylindrical and cuboid samples. On the other hand, as reported in [10], PCL started to crystallize at a relatively higher speed at 37 °C. Hence, it was practically very difficult to ensure that the programming condition was exactly the same every time. Thus, the seemingly slight fluctuation in shape fixity ratio in the strip samples might be the result of this.

**Figure 2:**
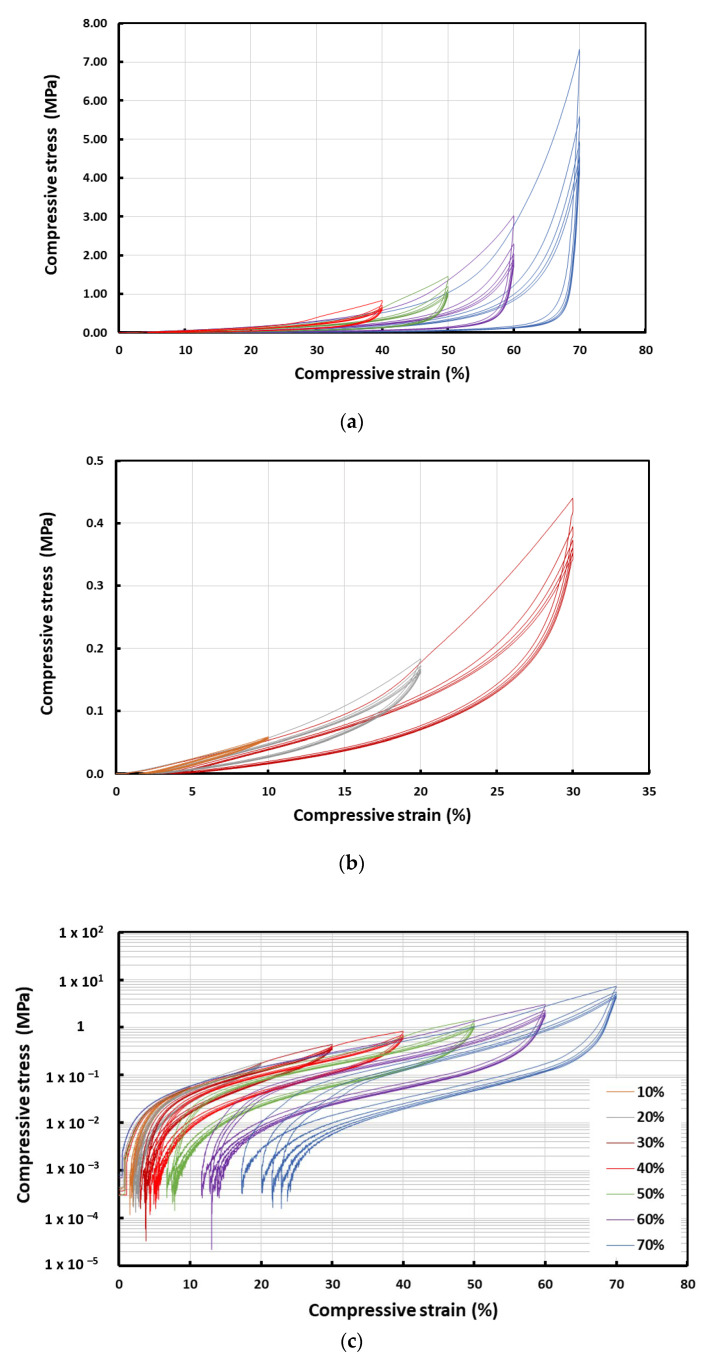
Typical stress vs. strain curves of cylindrical sample in cyclic uniaxial compression at room temperature (**a**) from 10% to 70% strain with an increment of 10% at each step; (**b**) zoomed in portions of the initial part of the curves at 10%, 20% and 30% strains; (**c**) logarithmic curves showing the residual strains.

Figure 2a shows the typical stress vs. strain curves of the cylindrical samples during uniaxial compression at room temperature from 10% to 70% strain with an increment of 10% at each step. Five cycles were performed at each strain. Figure 2b presents the zoomed in portions of the initial part of the curves at 10%, 20% and 30% strains. It can be observed that, for any compression strain, the stress values, as well as the residual strain, were the highest for the first cycle and the subsequent cycles had slightly lower stress values and were similar to each other. Hence, the Mullins effect was observed in these samples. Furthermore, as shown in Figure 2c, the residual strain in the sample increased as the programming strain applied on the sample increased.

**Figure 3:**
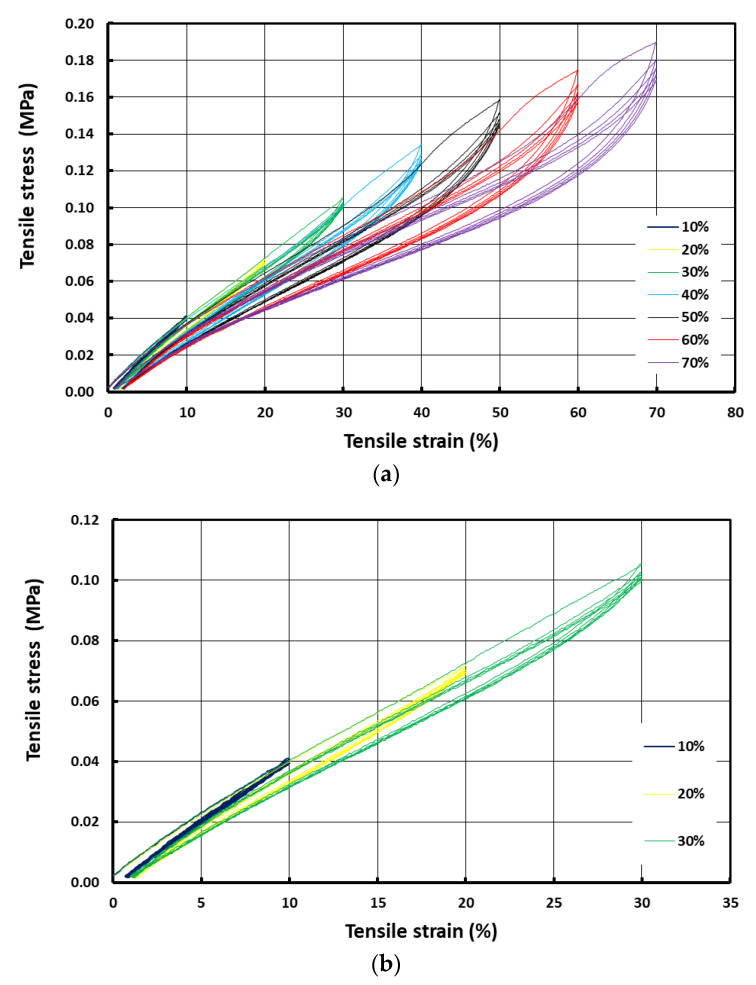
Typical stress vs. strain curves of strip sample in cyclic uniaxial tension at room temperature (**a**) from 10% to 70% strain with an increment of 10% at each step; (**b**) zoomed in portions of the initial part of the curves at 10%, 20% and 30% strains.

Figure 3a shows the stress vs. strain curves for strip samples subjected to cyclic tension at room temperature from 10% to 70% strains with an increment of 10% at each step. Figure 3b presents the zoomed in portions of the initial part of the curves at 10%, 20% and 30% tensile strains, respectively. Similar to the above samples subjected to cyclic compression, these strip samples also exhibited higher stress values for the first cycle and the subsequent cycles had slightly lower stress values that were similar to each other. Thus, it can be concluded that the Mullins effect was also observed in the strip samples.

For a particular type of hybrid, even if the total amount of inclusions (in volume percentage) was fixed, the actual stress vs. strain response upon loading might not be the same. As investigated in Supplementary Section S1, finite element simulation (assuming that the bonding between inclusions and matrix was strong, for simplicity) revealed that for the same volume percentage of spherical inclusions (assumed to be in the same size, for simplicity), the hybrids might have slightly different stress vs. strain response upon loading. The variation was more significant with decrease in the volume fraction of the inclusions. On the other hand, the variation was more in hybrids with harder inclusions. Hence, it was also expected that the hardness of the different samples and even of the different surfaces of the same sample could be slightly different.

**Figure 4:**
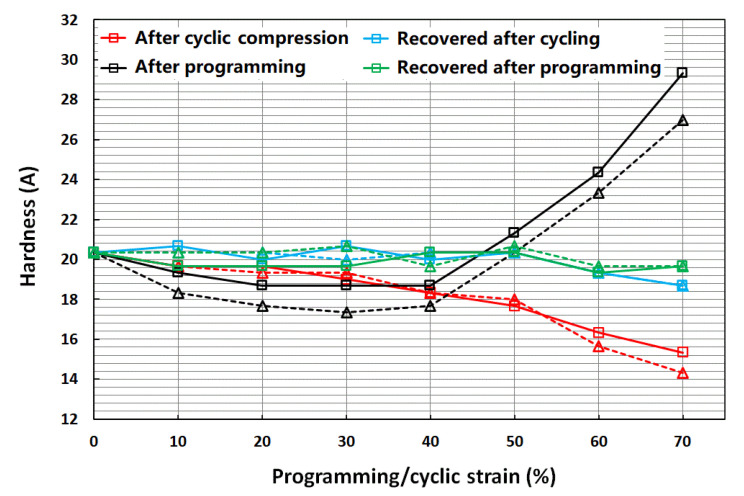
Shore hardness (average) of the top surface of two cylindrical samples (same color) at four different types of tests.

Figure 4 shows the evolution of hardness on the top surface (where the samples were compressed in the machine) of the cylindrical samples recorded for four different cases:

- After subjection to cyclic compression tests at 10, 20, 30, 40, 50, 60 and 70% strains;
- After the recovery of the samples that were subjected to cyclic compression in i;
- After subjection to programming tests at 10, 20, 30, 40, 50, 60 and 70% strains;
- After the recovery of the samples that were subjected to programming in iii.

As shown in Figure 4, after cyclic compression (red lines), we can see that the hardness of the samples dropped continuously after cyclic compression. The higher the applied compression strain, the more the reduction in the measured hardness. At the end of 70% cyclic strain, the hardness had dropped by about 27% from its initial value for the samples. However, once these samples were heated for recovery (blue lines), the hardness values of these samples almost fully reset to their initial values.

On the other hand, after programming (black lines), the hardness dropped by about 10% in the samples with 20–40% programming strains, and then increased rapidly for subsequent higher strains. The hardness increased by about 35–45% from initial values for the 70% programming strain. After they were heated for recovery (green lines), the hardness values of these samples almost fully reset to their initial values, similar to the hardness of the samples heated for recovery after cyclic compression tests, as discussed above.

**Figure 5:**
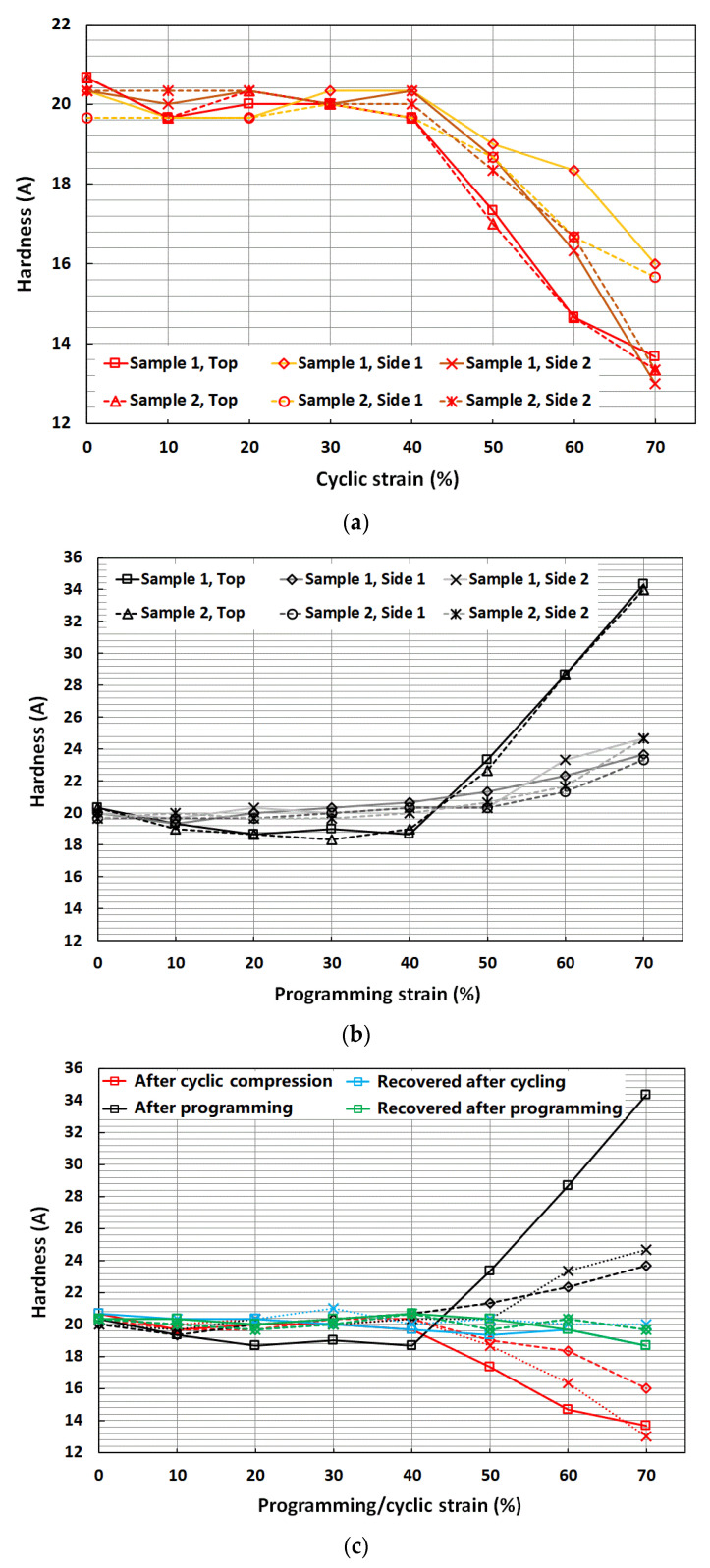
Shore hardness (average) of two cuboid samples (same color) on three different sides (**a**) after cyclic compression on the top surface; (**b**) after programming (compression) on the top surface. (**c**) Comparison of four different types of tests (results of one representative sample. Solid line: Top surface; dashed line: Side 1; dotted line: Side 2).

Figure 5a presents the evolution of hardness on three sides of the cuboid samples after cyclic compression. The red lines are for the hardness measured on the top side where the samples were compressed. The orange and brown lines are for the hardness measured on the left (Side 1) and right (Side 2) sides of the cuboid samples with respect to the top side. As observed, the hardness remained almost the same for up to 40% cyclic strain for all three sides and then started to decrease for subsequent higher strains. At the end of 70% cyclic strain, the hardness had dropped by about 34.6% for the top side and by an average of about 28% for Sides 1 and 2, respectively. However, after recovery, the hardness values reset to their initial values, as shown in Figure 5c (blue lines). The difference between the two samples was not significant.

Figure 5b shows the evolution of hardness on three sides of the cuboid samples after programming (compression). While the hardness of Sides 1 and 2 increased monotonically with increase in programming strain, it was only slight at small programming strains (less than 40%), and more significant at high programming strains (over 50%). The hardness of the top side (black lines) dropped by about 7% till 40% programming strain and then started to increase as the programmed strain increased. At 70% programming strain, the hardness had increased by about 68% on the top surface, while the increase was only about 21.4% on Sides 1 and 2. After heating for recovery (green lines in Figure 5c), we could see that the hardness values of these samples reset to their initial values, similar to cylindrical samples. Again, the difference between the two samples was small.

In Figure 5c, we summarize the hardness of all tests (including recovered samples after cycling and programming) using the results of one sample, since the difference between two samples was not much. It may be concluded that:

Cyclic compression remarkably reduced the hardness of all surfaces, if the applied compressive strain was over 40%;

Programming (via compression) increased the hardness in the transverse sides but the resulting hardness of the top (compressed) surface slightly decreased for small programming strains and increased for high programming strains.

**Figure 6:**
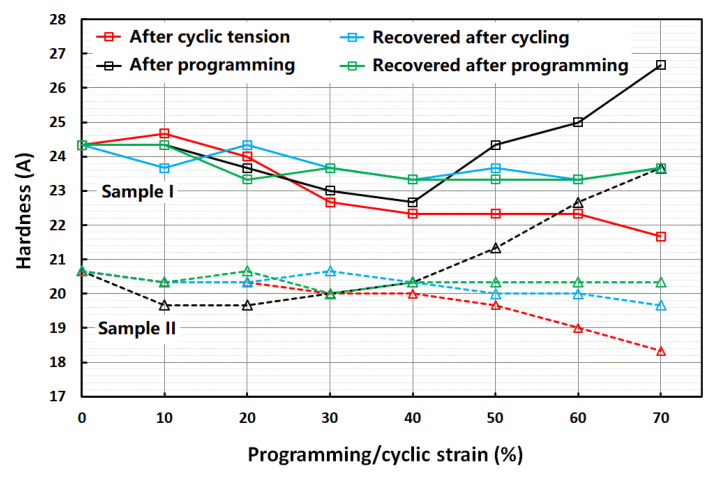
Shore hardness (average) of the top surface of strip samples. Red: after cyclic tension; blue: recovered after cycling; black: after programming; blue: recovered after programming.

Figure 6 shows the evolution of hardness of two strip samples (Sample I and Sample II) recorded for four different cases: after cyclic tension at 10, 20, 30, 40, 50, 60 or 70% strain; after recovery from cyclic tension; after programming with 10, 20, 30, 40, 50, 60 or 70% tensile strain; after recovery from programming. For both samples, the hardness trends were similar. For instance, upon cyclic tension, their hardness dropped with increase in cyclic strain. Around 11% drop in hardness had resulted at the end of 70% cyclic strain. After recovery, the hardness values reset to their initial values for both samples. For the programming test, the hardness dropped slightly till 40% programming strain and then started to increase as the programming strain increased further. At the end of 70% programming strain, the hardness had increased by about 15% and 10% for Samples I and II, respectively. After recovery, the hardness values once again reset to their initial values.

When we compare Figure 4 and Figure 6 for loading by tension and compression, respectively, the trends look similar: with increase in the strain applied in cyclic loading, the material became softer; and after heating for recovery, the material recovered its hardness.

However, if we further take Figure 5b into consideration, the influence of programming on hardness appeared to be not only dependent on the sample surface and mode of loading, but also dependent on the programming strain. Figure 5b shows that while the hardness on the (side) surface (perpendicular to the compression direction) increased with the increase in programming strain, the hardness on the top surface, where the compressive loading was applied, decreased and then increased with the increase in programming strain. On the other hand, if the applied programming loading was tension, the hardness on the (side) surface (perpendicular to the tension direction) decreased and then increased with the increase in programming strain.

## 4. Mechanisms for Evolution of Hardness

In Section 3, it was observed that:

(a) With the increase in the strain applied in cyclic loading, the material became softer.

(b) The influence of programming on hardness appeared to be not only sample surface and mode of loading dependent, but also programming strain dependent.

(c) After heating for recovery, the hardness recovered.

While (c) was apparently the result of excellent shape memory effect, further investigation was required for (a) and (b).

### 4.1. Debonding upon Loading

As mentioned in [2,5,15], a weak bonding between the matrix and inclusions is preferred in shape memory hybrids and, in particular, in elastic shape memory hybrids.

If the bonding between silicone and PCL is not strong, the observed downward trajectory in hardness in those samples undergoing cyclic loading should be the result of debonding. In Section S2 of Supplementary Materials, debonding upon stretching was numerically investigated via finite element analysis. A thin layer of interphase (with different Young’s modulus) between the spherical inclusion and matrix was used to examine the influence of different bonding strengths.

As revealed in Figure S12 in Supplementary Materials, with decrease in the Young’s modulus of the interphase, the maximum equivalent stress dropped, while the eye-ball phenomenon reported in the experiment (e.g., in [15]) became more and more significant. Consequently, in the corresponding stress vs. strain curves (Figure S13a in Supplementary Materials), the hybrid appeared to soften earlier. If the inclusion was elastic polylactic acid (PLA) (Figure S13b in Supplementary Materials), which is softer than PCL, softening occurred at a lower tensile stress. Such softening essentially results in the Mullins effect.

If the applied strain was low, there was no debonding. The hybrid was highly elastic, and its hardness was roughly a constant. However, debonding appeared under higher strain loading or cyclic loading. Therefore, the hardness dropped (Figure 5a and Figure 6).

A high internal stress during loading migth5t induce plastic deformation in the inclusions. Consequently, the elasticity of the elastic shape memory hybrids deteriorated. This is the reason why hard spherical inclusions and weak bonding are preferred in the design of elastic shape memory hybrids.

### 4.2. Hardness after Programming

As reported [9,10,15], after programming, elastic shape memory hybrids became harder. However, as revealed in Figure 5a and Figure 6, the hardness of the programmed shape memory hybrids might become softer or harder, depending on the surface and the programming strain.

**Figure 7:**
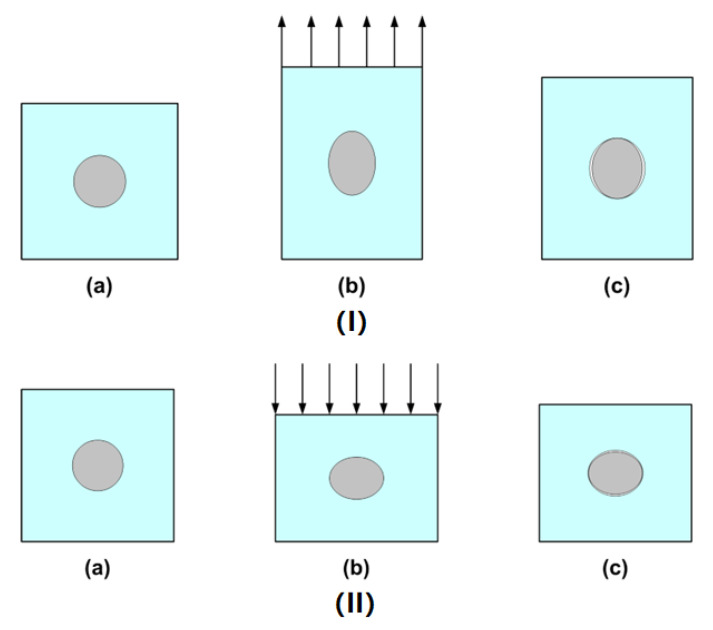
Illustration of structural change. (**a**) Original; (**b**) during programming; (**c**) after programming. (**I**) Programmed via tension. (**II**) Programmed via compression.

Figure 7 illustrates the structural change in a representative unit of a shape memory hybrid in a shape memory cycle. In this unit, a spherical inclusion (transition part) was embedded in a matrix (elastic part). While the inclusion was assumed to be hard at room temperature, the matrix was assumed to be always highly elastic. Upon heating to soften the inclusion, the spherical inclusion deformed into an elliptical shape. In the case of programming via stretching in the vertical direction (Figure 7(Ib)), the long axis of the elliptical inclusion was in the vertical direction, while in the case of programming via compression in the vertical direction (Figure 7(IIb)), the long axis of the elliptical shape (actually, it should be disc shape) was in the horizontal direction. After cooling to harden the inclusion, the applied load was removed, and the elastic matrix slightly recovered its original shape. In the case of weak bonding between the inclusion and matrix, debonding happened. Hence, a small gap was observed in the horizontal direction in the hybrid programmed via stretching (Figure 7(Ic)), or in the vertical direction if the hybrid was programmed via compression (Figure 7(IIc)).

The small gap softened the hybrid, and caused the hardness in the normal direction of the gap to decrease, while the hardness in the long axis direction of the elliptical inclusion increased. Since the matrix was elastic and the inclusion was hard, while the volume of the inclusion was fixed, if the programming strain was over a limit, which depended on the materials and the mode of programming, the hardness in the softened surface started to increase with increase in the programming strain. Consequently, the hardness of the programmed hybrids appeared to be dependent on not only the tested surface, but also the programming strain, exactly as reported in Figure 5a and Figure 6.

**Figure 8:**
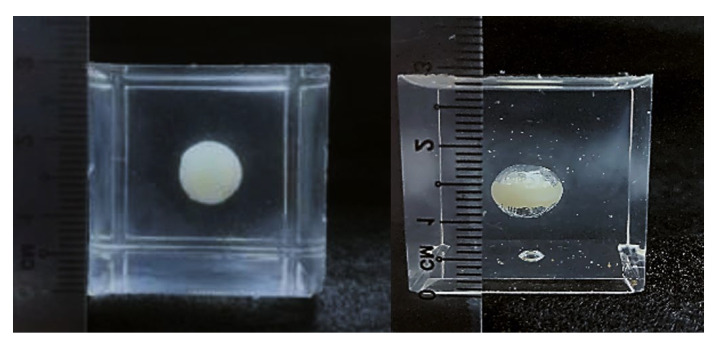
Spherical TPU inclusion embedded in silicone matrix before (**left**) and after (**right**) programming via compression in the vertical direction.

To demonstrate the formation of the gap after programming, we used another commonly used silicone, namely SylgardTM 184 Silicone Elastomer, (PDMS), as the matrix (size: 2.7 cm × 2.7 cm × 2.7 cm) and embedded a spherical inclusion (diameter: 0.7 cm) made of a thermoplastic polyurethane (TPU 265A) which has similar properties as the PCL used above (Figure 8, left). Refer to [15] and [56] for the properties of PDMS and TPU 265A, respectively. This sample was heated at 90 °C for 20 min to completely soften the inclusion, which had a melting temperature about 65 °C (refer to [56]), and then compressed to 20% of its gauge length (2.7 cm) at a constant speed of 2 mm/s. The compressed sample was cooled down to room temperature before unloading. Figure 8 (right) is the sample after programming, in which we can clearly see gaps above and underneath the elliptical shaped inclusion. Reheating to melt the inclusion again resulted in the sample reverting back to its original shape (the same as in Figure 8, left).

**Figure 9:**
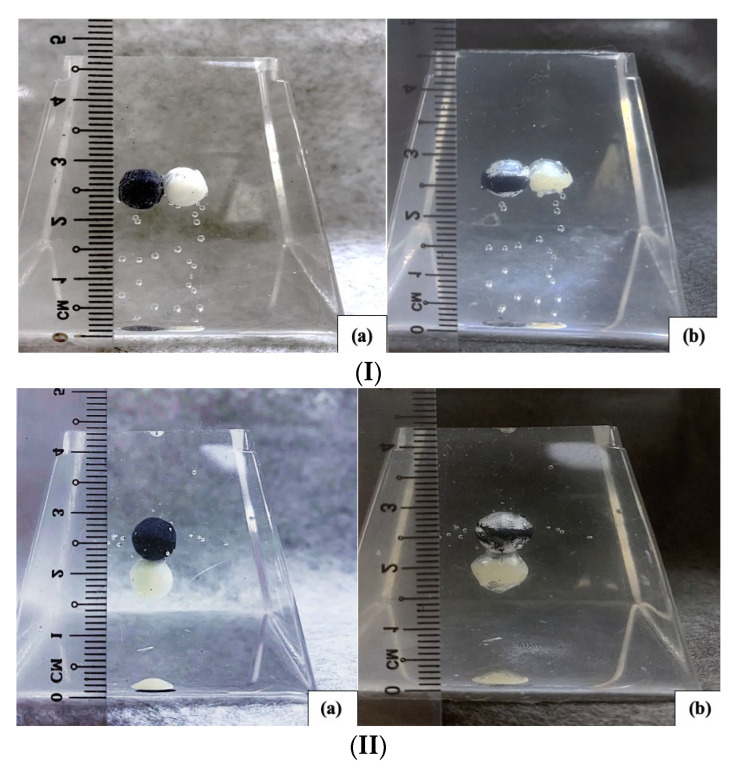
Two inclusions in contact with each other, before (**a**) and after (**b**) programming via high temperature compression in the vertical direction. (**I**) Two spherical inclusions packed horizontally; (**II**) two spherical inclusions packed vertically. The black inclusion was filled with carbon black.

If the volume fraction of inclusion was high (e.g., over 30 vol.%, as reported in [5]), some inclusions might be in direct contact. Two cases, in which two spherical inclusions (diameter: about 0.6 cm) were packed horizontally (Figure 9(Ia)) or vertically (Figure 9(IIa)) inside a PDMS block, were examined. The height of the PDMS blocks was 4.7 cm or 4.4 cm. After high temperature compression in the vertical direction by 20% of its initial height (0.94 or 0.88 cm), the inclusions might join together and gaps were observed in the top and bottom of all the elliptical shaped inclusions.

It appears that programming-induced gaps can provide a sound explanation for side/surface and programming mode-dependent hardness in programmed shape memory hybrids.

## 5. Further Discussion

Although we can see the huge potential of such elastic shape memory hybrids, in particular in body-temperature comfort fitting [9,10], there are some challenges faced in this field.

The first is to achieve about 100% shape fixity ratio, while maintaining high elasticity. This is difficult because the shape fixity ratio depends primarily on the percentage of transition components (PCL in this study) in the shape memory hybrid [5]. With the increase in the volume percentage of PCL, the resulting shape memory hybrid becomes harder and less elastic. Moreover, higher than 40% PCL (in volume) tends to form aggregates among the PCL inclusions, thereby losing the consistency and the uniformity of the hybrid. An elastic transition component that is of similar hardness to the elastic component can result in a high elastic shape memory hybrid at the cost of the shape fixity ratio.

The second challenge comes in the form of slightly sticky surfaces on the hybrid. This generally happens when a softer silicone is used as the elastic component. Softer silicones usually have silicone oil in them during their manufacturing process to make them softer (Shore hardness < 10 A). Upon using such silicones to fabricate shape memory hybrids, we noticed that the silicone oils became dispersed on the surface of the final fabricated hybrid, thereby giving a slightly sticky texture. Research is being carried out on finding an effective way to remove the stickiness without affecting the surface properties of the hybrid.

In this study, simulations were carried out on a basic level with the sole intention of revealing the key features in the theoretical behavior of shape memory hybrids when different inclusions were used. Similarly, simulations were performed to showcase the differences in the structure of inclusion and matrix when the interface between them was modelled in three ways (strong, medium and weak bonding), based on the assumption that the effect of the loading rate is negligible. Since the soft silicone we used in the course of this study is viscoelastic, the strain rate effect should be considered for more accurate simulation.

## 6. Conclusions

In this paper, the shape fixity ratio and shape recovery ratio of a body-temperature programmable elastic SMH was investigated. It was found that while the shape recovery ratio was always 100% in all samples, the shape fixity ratio increased with increase in programming strain in cases where that programming was via compression. A shape fixity ratio of about 90% was achievable when the applied compression programming strain was 40% or more. However, if programming was via tension, the resulting shape fixity ratio was always around 90%. Non-uniformity in temperature in the tested samples and slight inconsistency in testing conditions might be the reasons for such differences. The Mullins effect was confirmed in the samples subjected to cyclic loading in both uniaxial tension and uniaxial compression.

The evolution of shore hardness in cyclic uniaxial stretching or cyclic uniaxial compression to different strains, programming via tension or compression to different strains, and heating for recovery were systematically investigated.

On the one hand, cyclic loading (both tension and compression) remarkably reduced the hardness of the sample if the cyclic strain is over 30%. After heating to above the melting temperature of PCL, which was the material for the inclusions, the hardness of the samples mostly recovered.

On the other hand, the influence of programming on hardness depended on the type of loading in programming and the surface to be tested after programming. Instead of softening in cyclic loading, hardening was expected after programming. However, for certain surfaces, experimentally demonstrated here, the formation of a micro gap between the inclusion and matrix occurred after programming, and the hardness vs. programming relation was not monotonic, but decreased slightly and then increased dramatically. This was the same as the samples subjected to cyclic loading, as after heating for shape recovery, the hardness of the samples mostly returned.

Finite element simulation revealed that the variation in the stress vs. strain relationship in SMHs with the same volume percentage of inclusions, but different distribution, decreased with increase in the volume percentage of the inclusion. Debonding between the inclusions and matrix is the reason for the Mullins effect.

## Figures and Tables

**Table 1:** Samples prepared.

Samples	Pictures	Dimensions	Tests
Cylindrical	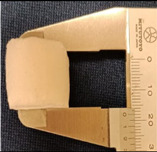	Diameter—1.8 cm Height—2 cm	1. Cyclic compression 2. Programming (compression)
Cuboid	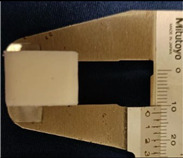	Length—2.7 cm Width—1.6 cm Height—1.2 cm	1. Cyclic compression 2. Programming (compression)
Strip	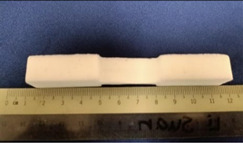	Length—3.2 cm Width—1.2 cm Thickness—0.5 cm	1. Cyclic tension 2. Programming (tension)

## Data Availability

The data that support the findings of this study are available from the corresponding author upon reasonable request.

## Supplementary Materials

The following supporting information can be downloaded at: https://www.mdpi.com/article/10.3390/polym14224872/s1.

## References

[1] Otsuka K., Wayman C.M. (1998). Shape Memory Materials.

[2] Huang W.M., Ding Z., Wang C.C., Wei J., Zhao Y., Purnawali H. (2010). Shape memory materials. Mater. Today.

[3] Sun L., Huang W., Ding Z., Zhao Y., Wang C., Purnawali H., Tang C. (2012). Stimulus-responsive shape memory materials: A review. Mater. Des..

[4] Huang W.M., Song C., Fu Y., Wang C., Zhao Y., Purnawali H., Lu H., Tang C., Ding Z., Zhang J. (2013). Shaping tissue with shape memory materials. Adv. Drug Deliv. Rev..

[5] Huang W.M., Zhao Y., Wang C.C., Ding Z., Purnawali H., Tang C., Zhang J.L. (2012). Thermo/chemo-responsive shape memory effect in polymers: A sketch of working mechanisms, fundamentals and optimization. J. Polym. Res..

[6] Fan K., Huang W.M., Wang C.C., Ding Z., Zhao Y., Purnawali H., Liew K.C., Zheng L.X. (2011). Water-responsive shape memory hybrid: Design concept and demonstration. Express Polym. Lett..

[7] Sun L., Huang W., Wang T., Chen H., Renata C., He L., Lv P., Wang C. (2017). An overview of elastic polymeric shape memory materials for comfort fitting. Mater. Des..

[8] Ahmad M., Luo J., Xu B., Purnawali H., King P.J., Chalker P.R., Fu Y., Huang W., Miraftab M. (2011). Synthesis and Characterization of Polyurethane-Based Shape-Memory Polymers for Tailored T-g around Body Temperature for Medical Applications. Macromol. Chem. Phys..

[9] Wang T.X., Renata C., Chen H.M., Huang W.M. (2017). Elastic shape memory hybrids programmable at around body-temperature for comfort fitting. Polymers.

[10] Naveen B.S., Naseem A.B.M., Ng C.J.L., Chan J.W., Lee R.Z.X., Teo L.E.T., Wang T., Nripan M., Huang W.M. (2021). Body-Temperature Programmable Soft-Shape Memory Hybrid Sponges for Comfort Fitting. Polymers.

[11] Zhu G., Xu S., Wang J., Zhang L. (2006). Shape memory behaviour of radiation-crosslinked PCL/PMVS blends. Radiat. Phys. Chem..

[12] Salvekar A.V., Zhou Y., Huang W.M., Wong Y.S., Venkatraman S.S., Shen Z., Zhu G., Cui H.P. (2015). Shape/temperature memory phenomena in un-crosslinked poly-ɛ-caprolactone (PCL). Eur. Polym. J..

[13] Chern M.-J., Yang L.-Y., Shen Y.-K., Hung J.-H. (2013). 3D scaffold with PCL combined biomedical ceramic materials for bone tissue regeneration. Int. J. Precis. Eng. Manuf..

[14] Wang T.X., Huang W.M., Aw J.E., He L.W., Vettorello M. (2017). Comfort fitting using shape memory polymeric foam. J. Test. Eval..

[15] Wang C.C., Huang W.M., Ding Z., Zhao Y., Purnawali H., Zheng L., Fan H., He C.B. (2012). Rubber-like shape memory polymeric materials with repeatable thermal-assisted healing function. Smart Mater. Struct..

[16] Mullins L. (1969). Softening of rubber by deformation. Rubber Chem. Technol..

[17] Diani J., Fayolle B., Gilormini P. (2009). A review on the Mullins effect. Eur. Polym. J..

[18] Amin A., Alam M., Okui Y. (2002). An improved hyperelasticity relation in modeling viscoelasticity response of natural and high damping rubbers in compression: Experiments, parameter identification and numerical verification. Mech. Mater..

[19] Rickaby S., Scott N. (2013). Cyclic stress-softening model for the Mullins effect in compression. Int. J. Non-Linear Mech..

[20] Beatty M.F. (2000). The Mullins Effect in a Pure Shear, in Advances in Continuum Mechanics and Thermodynamics of Material Behavior.

[21] Plagge J., Klüppel M. (2019). Mullins effect revisited: Relaxation, recovery and high-strain damage. Mater. Today Commun..

[22] Dorfmann A. (2003). Stress softening of elastomers in hydrostatic tension. Acta Mech..

[23] Ragni L., Tubaldi E., Dall’Asta A., Ahmadi H., Muhr A. (2018). Biaxial shear behaviour of HDNR with Mullins effect and deformation-induced anisotropy. Eng. Struct..

[24] Mars W., Fatemi A. (2004). Observations of the constitutive response and characterization of filled natural rubber under monotonic and cyclic multiaxial stress states. J. Eng. Mater. Technol..

[25] Machado G., Chagnon G., Favier D. (2010). Analysis of the isotropic models of the Mullins effect based on filled silicone rubber experimental results. Mech. Mater..

[26] Mai T.-T., Morishita Y., Urayama K. (2017). Induced anisotropy by Mullins effect in filled elastomers subjected to stretching with various geometries. Polymer.

[27] Machado G., Chagnon G., Favier D. (2012). Induced anisotropy by the Mullins effect in filled silicone rubber. Mech. Mater..

[28] Fu W., Wang L., Huang J., Liu C., Peng W., Xiao H., Li S. (2019). Mechanical properties and Mullins effect in natural rubber reinforced by grafted carbon black. Adv. Polym. Technol..

[29] Rault J., Marchal J., Judeinstein P., Albouy P.A. (2006). Stress-induced crystallization and reinforcement in filled natural rubbers: 2H NMR study. Macromolecules.

[30] Kitey R., Upadhyay C. (2022). Effect of Carbon Black Content on Quasi-Static Compression Behaviour of Filled Rubber, in Aerospace and Associated Technology.

[31] Allen V., Chen L., Englert M., Moussaoui A., Pisula W. (2021). Control of Mullins stress softening in silicone elastomer composites by rational design of fumed silica fillers. Compos. Sci. Technol..

[32] Persson A.-M.M.R., Andreassen E. (2022). Cyclic Compression Testing of Three Elastomer Types—A Thermoplastic Vulcanizate Elastomer, a Liquid Silicone Rubber and Two Ethylene-Propylene-Diene Rubbers. Polymers.

[33] Krpovic S., Dam-Johansen K., Skov A.L. (2021). Importance of Mullins effect in commercial silicone elastomer formulations for soft robotics. J. Appl. Polym. Sci..

[34] Dorfmann A., Ogden R.W. (2004). A constitutive model for the Mullins effect with permanent set in particle-reinforced rubber. Int. J. Solids Struct..

[35] Zhu P., Zhong Z. (2021). Constitutive modelling for the mullins effect with permanent set and induced anisotropy in particle-filled rubbers. Appl. Math. Model..

[36] Boyce M.C., Kear K., Socrate S., Shaw K. (2001). Deformation of thermoplastic vulcanizates. J. Mech. Phys. Solids.

[37] Maiti A., Small W., Gee R.H., Weisgraber T.H., Chinn S.C., Wilson T.S., Maxwell R.S. (2014). Mullins effect in a filled elastomer under uniaxial tension. Phys. Rev. E.

[38] Srivastava S.K., Mishra Y.K. (2018). Nanocarbon reinforced rubber nanocomposites: Detailed insights about mechanical, dynamical mechanical properties, payne, and mullin effects. Nanomaterials.

[39] Houwink R. (1956). Slipping of molecules during the deformation of reinforced rubber. Rubber Chem. Technol..

[40] Rigbi Z. (1980). Reinforcement of rubber by carbon black. Properties of Polymers.

[41] Corby M., de Focatiis D.S. (2019). Reversibility of the Mullins effect for extending the life of rubber components. Plast. Rubber Compos..

[42] Yan L., Dillard D.A., West R.L., Lower L.D., Gordon G.V. (2010). Mullins effect recovery of a nanoparticle-filled polymer. J. Polym. Sci. Part B Polym. Phys..

[43] Wang S., Chester S.A. (2018). Modeling thermal recovery of the Mullins effect. Mech. Mater..

[44] Sun Y., Yang L., Liu F., Wang Z. (2020). Mullins Effect and Its Reversibility for Thermoplastic Vulcanizates Based on Ethylene–Acrylic Acid Copolymer/Nitrile–Butadiene Rubber Blends. Polym. Sci. Ser. A.

[45] Zhao J., Wang C., Wang Z. (2015). Mullins effect and its reversibility of compatibilised thermoplastic vulcanisates based on high impact polystyrene/high vinyl polybutadiene rubber blend. Plast. Rubber Compos..

[46] Wineman A., Shaw J. (2007). Combined deformation-and temperature-induced scission in a rubber cylinder in torsion. Int. J. Non-Linear Mech..

[47] Harwood J., Payne A.R. (1966). Stress softening in natural rubber vulcanizates. Part III. Carbon black-filled vulcanizates. J. Appl. Polym. Sci..

[48] Zhao H., Allanson D., Ren X. (2015). Use of shore hardness tests for in-process properties estimation/monitoring of silicone rubbers. J. Mater. Sci. Chem. Eng..

[49] Qi H., Joyce K., Boyce M. (2003). Durometer hardness and the stress-strain behavior of elastomeric materials. Rubber Chem. Technol..

[50] Meththananda I.M., Parker S., Patel M.P., Braden M. (2009). The relationship between Shore hardness of elastomeric dental materials and Young’s modulus. Dent. Mater..

[51] Prem N., Schale F., Zimmermann K., Gowda D.K., Odenbach S. (2021). Synthesis and characterization of the properties of thermosensitive elastomers with thermoplastic and magnetic particles for application in soft robotics. J. Appl. Polym. Sci..

[52] Eshraghi S., Das S. (2010). Mechanical and microstructural properties of polycaprolactone scaffolds with one-dimensional, two-dimensional, and three-dimensional orthogonally oriented porous architectures produced by selective laser sintering. Acta Biomat..

[53] Wu X.L., Huang W.M., Lu H.B., Wang C.C., Cui H.P. (2017). Characterization of polymeric shape memory materials. J. Polym. Eng..

[54] Wu X., Huang W., Tan H. (2013). Characterization of shape recovery via creeping and shape memory effect in ether-vinyl acetate copolymer (EVA). J. Polym. Res..

[55] Sun L., Huang W.M., Wang C.C., Zhao Y., Ding Z., Purnawali H. (2011). Optimization of the shape memory effect in shape memory polymers. J. Polym. Sci. Part A Polym. Chem..

[56] Sun L., Huang W.M., Lu H., Lim K.J., Zhou Y., Wang T.X., Gao X.Y. (2014). Heating-responsive shape-memory effect in thermoplastic polyurethanes with low melt-flow index. Macromol. Chem. Phys..

