# Supplementary material for: Evolution of Shore Hardness under Uniaxial Tension/Compression in Body-Temperature Programmable Elastic Shape Memory Hybrids
Source: Polymers (Basel). 2022 Nov 11;14(22):4872. doi: 10.3390/polym14224872 (PMC9697891; doi:10.3390/polym14224872)
Supplement: Supplementary file 1 [file polymers-14-04872-s001.zip › polymers-1951502-supplementary.pdf]

polymers

MDPI

Supplementary material

Evolution of Shore Hardness under Uniaxial Tension/Compression in Body-Temperature Programmable Elastic Shape Memory Hybrids

Balasundaram Selvan Naveen [1], Nivya Theresa Jose [2], Pranav Krishnan [3], Subham Mohapatra [4], Vivek Pendharkar [5], Nicholas Yuan Han Koh [1], Woon Yong Lim [1] and Wei Min Huang [1,*]

[1] School of Mechanical and Aerospace Engineering, Nanyang Technological University, 50 Nanyang Avenue, Singapore 639798, Singapore
[2] Polymer Science and Engineering, Indian Institute of Technology, Roorkee, 247667, India
[3] Department of Metallurgical and Materials Engineering, Indian Institute of Technology, Kharagpur 721302, India
[4] Department of Mechanical Engineering, National Institute of Technology, Rourkela 769008, India
[5] Department of Metallurgical Engineering and Materials Science, Indian Institute of Technology, Bombay, Powai, Mumbai 400076, India
* Correspondence: mwmhuang@ntu.edu.sg

S1 Variation in stress vs. strain response of hybrids

The simulations performed in this section were modelled based on the assumption that the bonding between the silicone matrix and the inclusions were perfect (i.e., there was no debonding effect). The inclusions considered for this study are polycaprolactone (PCL) and elastic polylactic acid (PLA) (OYU-PLA, from Daiso, Hiroshima, Japan), and the silicone considered is E620 from Hong Ye Jie Technology Co., Ltd., Shenzhen, China (Shore hardness: 20A). The simulations were performed to investigate the influence of:

- Inclusion distribution (at fixed volume fraction of inclusion);
- Volume fraction of the inclusion (size of inclusions fixed);
- Different inclusion material (PCL or PLA).

on the stress vs. strain response in tension and compression of the shape memory hybrids.

The hybrid material design was done using MSC Digimat®(by Exstream, Digimat 2019.1, Mont-Saint-Guibert, Belgium). The meshing and loading parameters were input and the file was exported as a Python script to ANSYS Workbench 2019. The results were evaluated and visualised using ANSYS Mechanical (Ansys 2021 R2 (21.2), ANSYS, Canonsburg, USA). A linear elastic model was used for the matrix, while an elastoplastic model was taken for the randomly distributed inclusions. It was assumed that the stress vs. strain response was independent of the strain rate applied during loading. The hybrid was loaded in uniaxial tension or compression. The finite element analysis was solved with the Dirichlet boundary condition (displacement type loading). The material properties are tabulated as shown in Table S1. The values for PCL were obtained from [1].

Table S1. Material properties used in simulation (data collected from [1,2]).

Material	Young's modulus (MPa)	Poisson's ratio	Yield strength (MPa)	Ultimate strength (MPa)	Hardening modulus (MPa)	Hardening exponent	Linear modulus (MPa)
PCL (tension)	363.4	0.3	8.2	10.5	4.2	325	-
PCL (compression)	297.8	0.3	12.5	38.7	8	320	100
PLA	30	0.36	1.5	2.4	1	50	0.2
Silicone	0.42	0.47	0.2	0.4	-	-	-

The key parameters used in this study are as follows:

- Periodic geometry;
- Interpenetration of inclusions NOT allowed;
- Minimal relative distance between inclusions = 0.05;
- Random algorithm seed automatically selected.

As we can see in the details above, periodic geometry was considered, as it avoided any bias in the form of interpenetration of inclusions or coatings, etc. User-defined volume fraction was used as opposed to the maximum packing algorithm, as the parameter to be used while creating the representative elementary volume (RVE) (cube size: 1 m × 1 m × 1 m). The automatic random algorithm seed ensured that the inclusions were evenly distributed in a random manner, which was different every time a simulation was performed. There was no overlap between the inclusions. All phases (here, the matrix and inclusion) were generated simultaneously. The diameter of every inclusion was ensured to be the same by toggling the number of inclusions per RVE, to obtain the desired volume fraction. The size ratio of the inclusion diameter to the RVE edge length was 0.15:1, unless specified otherwise. Hence, the diameter of the inclusion was 0.15 m.

A conforming (tetra) type of mesh was used in which the element size and minimum element size parameters were automatically decided based on the volume fraction and number of inclusions which were given as input parameters. Quadratic elements helped for a finer control of geometry; however, internal coarsening was required to ensure that the distance of inclusions to bounding faces and edges remained more or less constant. Curvature control helped in adjusting sizes of curved inclusions. Five refinement steps were taken to prevent the computation from being too expensive. With this selected mesh, the RVE was divided into about 50,000 elements in each finite element simulation.

Uniaxial loading along the X-axis—in tension or compression—was applied in this study. For this particular type of loading, the Dirichlet boundary condition (displacement type loading) was used instead of periodic boundary conditions, since it saved significantly on the total computational cost and time required to do the analysis.

For finite element analysis, this type of boundary condition was applied using multi-point kinematic constraints between the eight corner nodes of the RVE. These eight corner nodes acted as the control nodes which were acted upon by displacement fields depending on the type of loading, peak strains and loading history. For all nodes lying on each of the six faces of the RVE, the displacement fields were not controlled directly, but were interpolated using the multi-point kinematic constraints applied on the eight corner nodes. For this case, the interpolation for a particular face of the RVE was done in a bilinear manner using the four corner nodes of that face. A similar procedure was followed for all of the six faces. Therefore, the displacement of the RVE was measured relative to these corner nodes.

Periodic boundary conditions on the other hand were used when the flux of the field variable (which is displacement in this case) was ensured to remain periodic with respect to all faces of the RVE. A large number of equations came about which related the degrees of freedom of nodes on one face with those of the corresponding nodes lying on its

opposite face. At times, non-periodic meshes happened for which node duplication was done to maintain periodicity of the flux of the field variable.

Periodic boundary conditions provided the best results but involved a huge number of equations involving all nodes, whereas Dirichlet boundary conditions provided close to desirable results with lesser CPU time and computational costs.

The loading source used was Digimat itself, which implied that all loading parameters were user-defined. The loading used was quasi-static loading (strain-controlled), since the stress vs. strain response was independent of strain rate. The samples in tension were loaded up to 50% strain, whereas those in compression were loaded up to 30%.

Finally, the file was imported into ANSYS Workbench as a python script and ANSYS Mechanical was used to solve the problem iteratively.

Three different meshing geometries were generated using a conforming tetrahedral mesh, while maintaining the same volume fraction of 20% between the matrix and the inclusion. The different geometries and distribution of inclusions are shown in Figure S1.

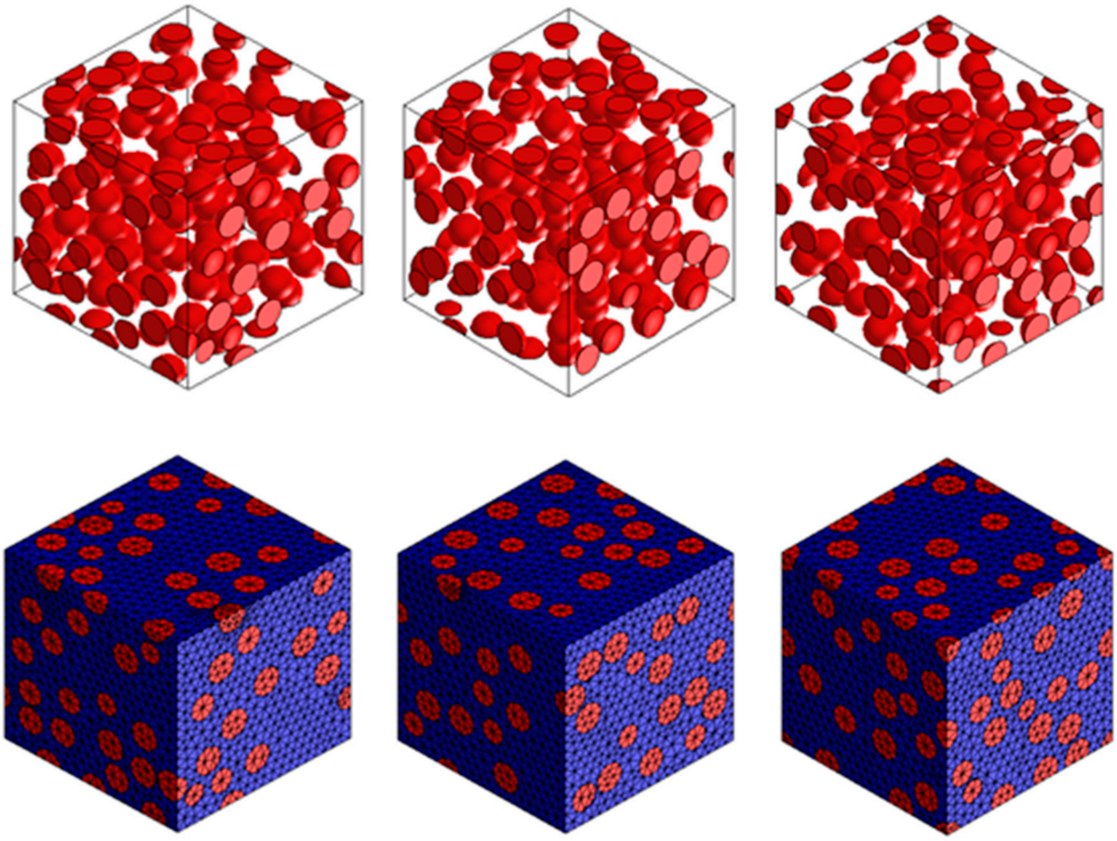

Figure S1. Typical distribution of inclusions (top row) and mesh (bottom row). (20 vol.%). Cube (hollow and blue colored) represents the matrix and the red colored spheres represent the inclusions.

On tensile loading, the RVE deformed longitudinally, and a stress distribution was developed in the geometry. In this particular example, PCL was the inclusion used. As it was about 1000 times stiffer than the matrix, it was naturally observed that the inclusions deformed less, and hence, were regions of higher stress. Minimal deformation of the inclusion could be observed from the inclusion-only stress distribution. It is further noticed that the inclusions at the RVE surface were distorted to a greater extent than those in the interior. This was due to the choice of boundary condition. The Dirichlet boundary condition was imposed to constrain the RVE. Field boundary condition was applied to all

the faces of the volume element to maintain the shape of the surfaces upon loading. This resulted in significantly more elongation at the surface. Loading was defined in terms of multipoint kinematic constraints, where the eight nodes at the corner of the volume element were used as control nodes on which displacement was applied. The same observation was made for the PLA inclusions (volume fraction = 0.3). Figure S2 and Figure S3 show typical stress distribution of PCL and PLA in tension, respectively. As PLA has a stiffness that is 10 times smaller than PCL, its maximum stress values are lower overall.

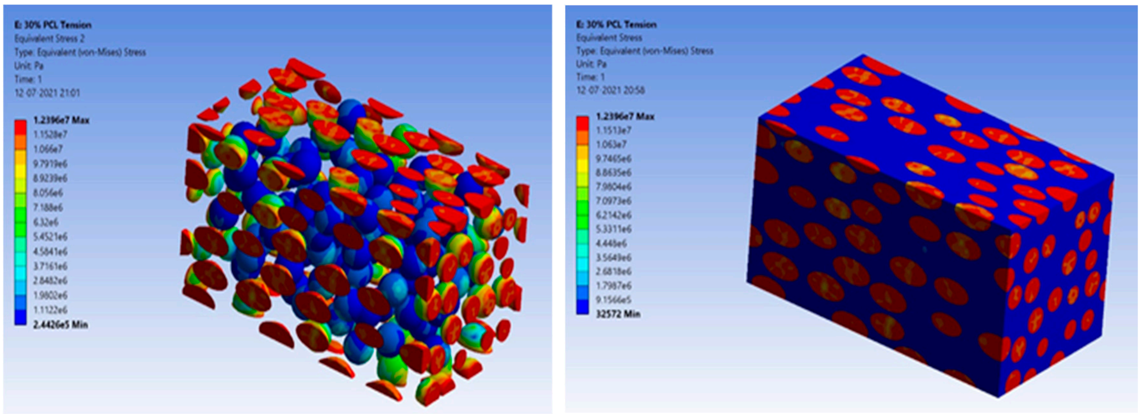

Figure S2. Typical stress distribution of PCL in tension (left: inclusions only; right: RVE).

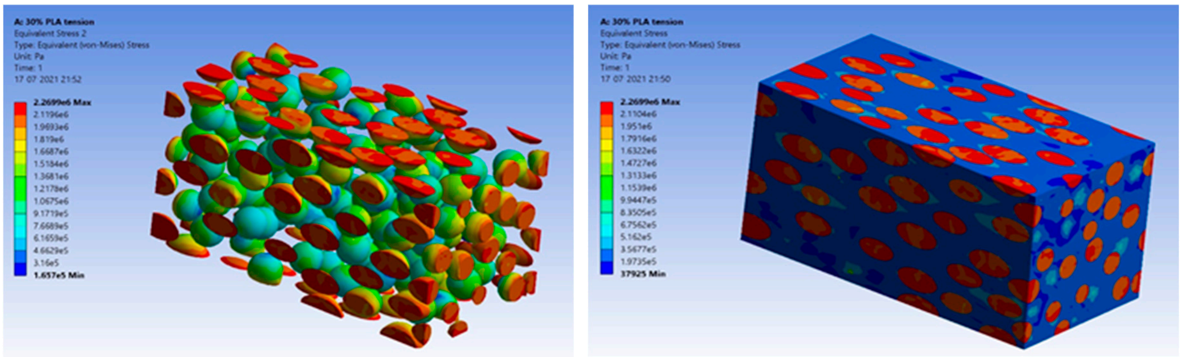

Figure S3. Typical stress distribution of PLA in tension (left: inclusions only; right: RVE).

In this study, three different volume fractions (VF) of 0.1, 0.2 and 0.3 were chosen to represent the volume ratios of inclusions to that of matrix. Five different simulations were run for each VF where each simulation had a different random distribution of inclusions in the matrix. To compare the range of variation with distribution, between the three VFs, a 'band plot' was constructed where the range is displayed as a coloured band for each VF. The same two inclusions (PCL and PLA) using in the previous sections were used here as well.

This study was extended to compressive loading, and a similar band plot is made out of five different distributions. Following these individual results, a couple of comparative studies were performed to visualise variation over a parameter in a single plot.

The following two comparative studies were analysed in detail:

1. 'Hard' (PCL) vs. 'Soft' (PLA) inclusion in the matrix.
2. The differing response of the silicone/PCL composite in tension and compression.

Figure S4 shows the stress vs. strain curves for silicone/PCL hybrids in tension. A maximum tensile strain of 50% was applied to the composite. It can be seen that, higher the VF of the inclusions, higher is the tensile stress induced in the sample. This in turn indicates that the sample with higher VF is harder. This is consistent with the fact that PCL is significantly harder than the silicone, given that the Young's modulus of PCL is

roughly 1000 times greater than that of silicone. A similar observation can also be made for PLA as well as seen in Figure S5.

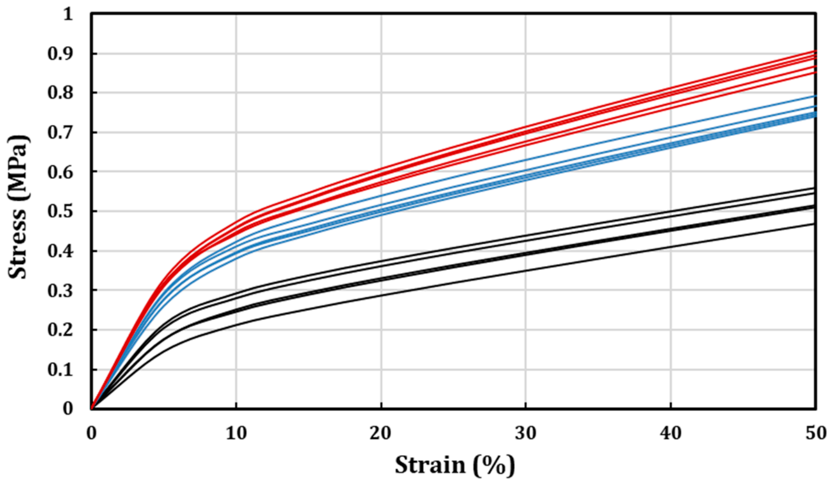

Figure S4. Band plot showing the width of variation of silicone/PCL hybrids in 0.3 VF (red), 0.2 VF (blue) and 0.1 VF (black) under tension.

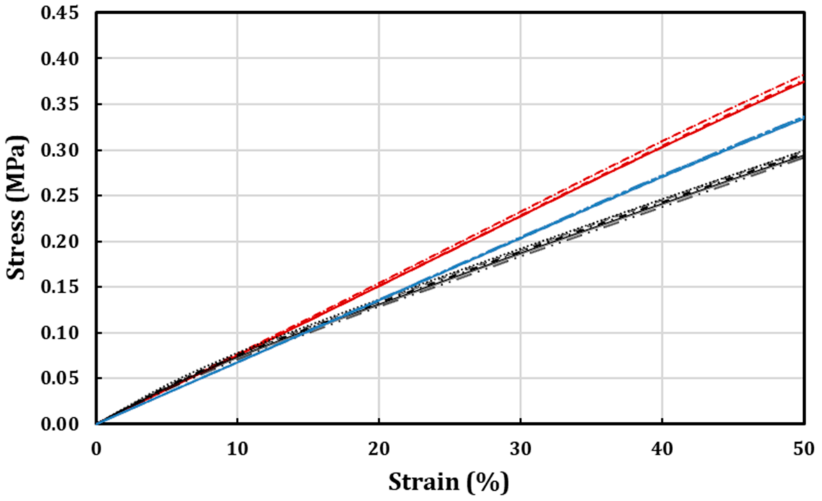

Figure S5. Band plot showing the width of variation of silicone/PLA hybrids in 0.3 VF (red), 0.2 VF (blue) and 0.1 VF (black) under tension.

Comparing the maximum stresses obtained at any particular strain for silicone/PCL and silicone/PLA hybrids, the silicone/PCL hybrids produced consistently higher stress values when compared with silicone/PLA hybrids. This also is consistent with the fact that PCL is a harder material than PLA.

Figure S6 shows the stress vs. strain relationship of silicone/PCL hybrids under compression loading.

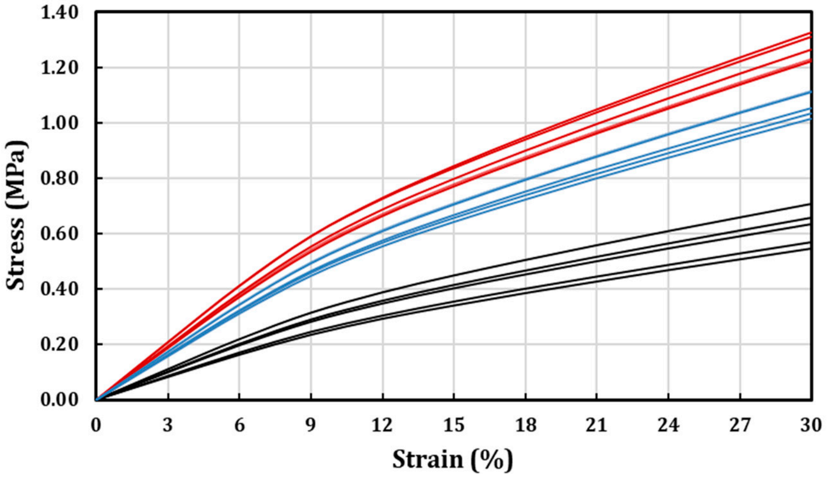

Figure S6. Band plot showing the width of variation of silicone/PCL material in 0.3 VF (red), 0.2 VF (blue) and 0.1 VF (black) under compression.

To analyse the effect of the distribution of inclusions in the matrix, coefficient of variation (CV) was calculated (standard deviation/mean) for each of the five random distribution cases at each strain value and then plotted together to observe the similarities or differences arising out of different distributions (while maintaining the same VF) of inclusions. Figure S7 shows the plots of CV against strain for PCL both in tension and compression, while Figure S8 is the plots of CV against strain for PCL and PLA in tension.

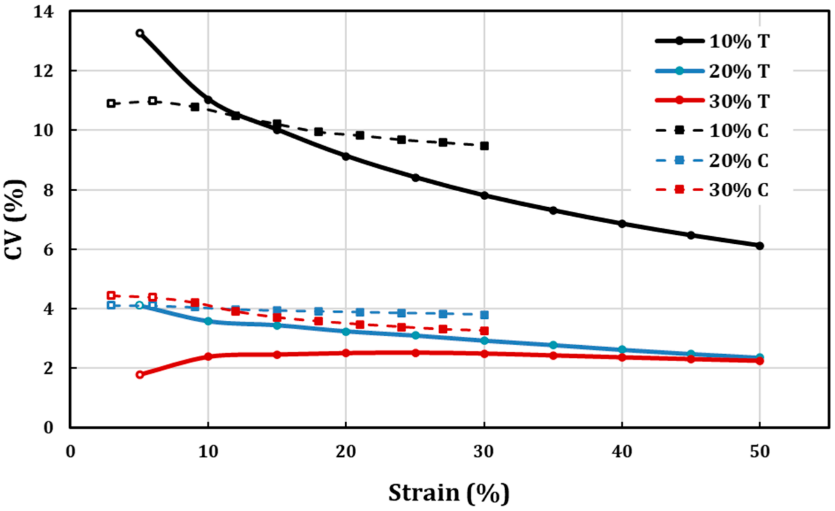

Figure S7. CV for silicone with PCL inclusions in tension (T) and compression (C) loading for different volume fraction (VF) of PCL. Continuous and dashed lines represent tension and compression, respectively, for 0.3 VF (red), 0.2 VF (blue) and 0.1 VF (black). 'Hollow' datapoints represent the composite being in the elastic region, whereas solid filled datapoints imply it has transitioned into the plastic region.

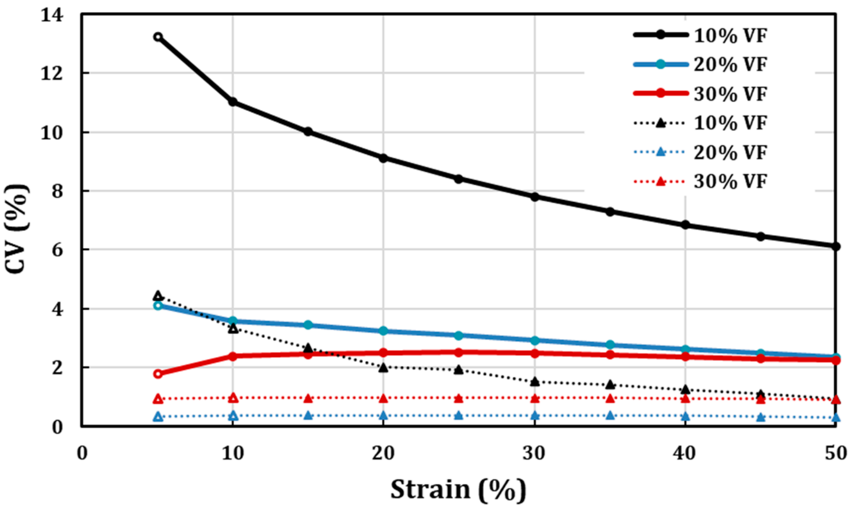

Figure S8. CV for silicone with different VF of PCL or PLA inclusions in tension. Circular and triangular data points represent PCL and PLA, respectively, for 0.3 VF (red), 0.2 VF (blue) and 0.1 VF (black). 'Hollow' datapoints represent the composite being in the elastic region, whereas solid filled datapoints imply it has transitioned into the plastic region.

The following trends can be observed from Figure S7 and Figure S8,

In general, CV decreases with the increase in VF and turns to become stable when the inclusions are loaded into the plastic range.

For the same VF, the CV of the hybrids with softer inclusions is much lower than that of hard inclusions.

In addition to numerical simulations, we have 3D printed a cube (3 cm × 3 cm × 3cm) by fused deposition modelling (FDM) and in the same as mentioned above to introduce inclusions into the hybrids, introduced randomly distributed spherical holes (diameter: 0.6 mm) in it with the total VF of 20% holes. The 3D printed cube made of Flexfill TPU 98A (100% infill) was printed using Creality Ender 3 V2 (Creality, Shenzhen, China). The designed model and the printed piece are presented in Figure S9. Each side of this cube was then subjected to cyclic compression loading to 0.5% at a strain rate of 10^{-4} /s using Shimadzu AG-10kNXplus STD (Shimadzu, Kyoto, Japan) universal testing machine. The obtained stress vs. strain curves are plotted in Figure S10, in which 1 and 1′, 2 and 2′, and 3 and 3′ are three pairs of opposite sides of the cube. Although this is an over simplified model, in which inclusions are replaced by holes, and FDM introduced some processing parameters in printing even we have set infill to 100%, the difference in response to compression for different sides is obvious. We can see a small but apparent difference in the slopes of opposite sides in the final cycle.

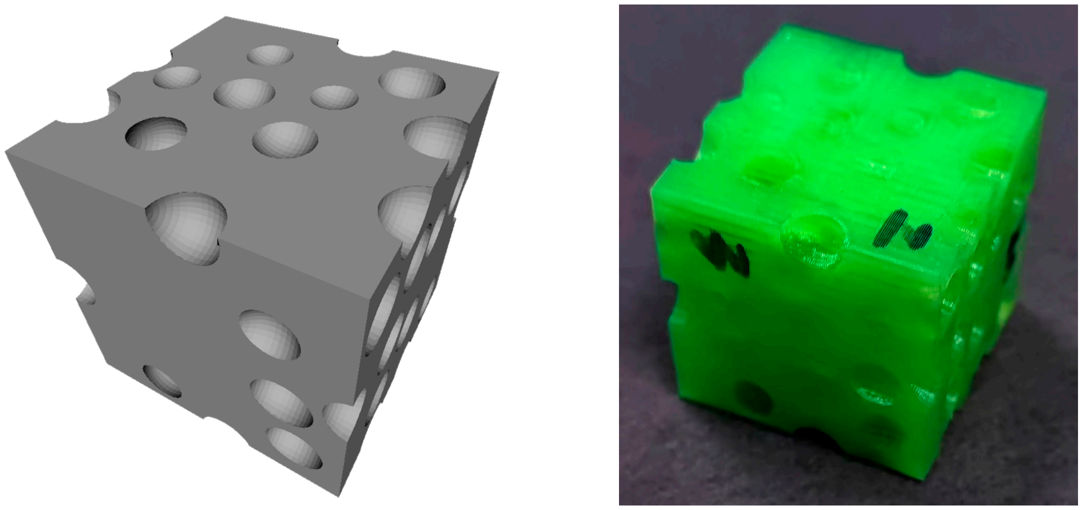

(**a**) (**b**)

Figure S9. Model (VF=0.2) (**a**) and 3D printed (**b**).

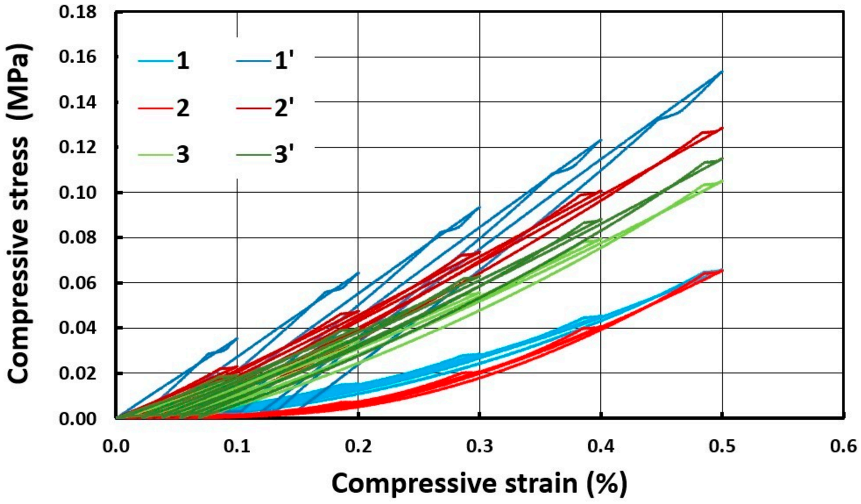

Figure S10. Stress vs. strain relationships under compression. 1 and 1′, 2 and 2′, and 3 and 3′ are pairs of opposite sides of the cube.

S2 Debonding between inclusion and matrix

In shape memory hybrids, in particular silicone based elastic shape memory hybrids, the bonding between the inclusions and matrix was very weak [3, 4]. Simulations were performed to have a theoretical understanding of the debonding between PCL inclusions and silicone matrix in a shape memory hybrid. Same as in Section S1, material design was done using MSC Digimat® (by Exstream), the meshing and loading parameters were input and the file was exported as a Python script to ANSYS Workbench 2019. The results were evaluated and visualized using ANSYS Mechanical.

A linear elastic model was used for the matrix, while an elastoplastic model was taken for the inclusion material. Additionally, the interphase material as well as the interface were assigned. The interphase material was modelled using a linear elastic model and had a Young's modulus which was 1/10th of that of the silicone matrix. For the interface, a cohesive model was used with a damage criterion in which maximum stress was used as the damage initiation criterion and displacement was used for the damage evolution law.

The hybrids were loaded in uniaxial tension up to 20% strain and the resulting stress vs. strain curves were plotted. The bonding cases were categorized on the basis of strength of the cohesive interface material, where for weak, medium and strong bonding, the elastic modulus of the interface is 1/100th, 1/10th and 1 times the Young's modulus of the silicone matrix, respectively. The materials used in simulation were E620 silicone for the matrix and PCL for the inclusion. The material properties of these materials are stated in Table S2.

Table S2. Key parameters for materials used in simulation.

<table>
<tr><th>Material</th><th colspan="2">Young's modulus (MPa)</th><th>Poisson's ratio</th><th>Yield strength (MPa)</th><th>Ultimate strength (MPa)</th><th>Hardening modulus (MPa)</th><th>Hardening exponent</th><th>Linear modulus (MPa)</th></tr>
<tr><td>PCL</td><td colspan="2">363.4</td><td>0.3</td><td>8.2</td><td>10.5</td><td>4.2</td><td>325</td><td>-</td></tr>
<tr><td>PLA</td><td colspan="2">30</td><td>0.36</td><td>1.5</td><td>2.4</td><td>1</td><td>50</td><td>0.2</td></tr>
<tr><td>Silicone</td><td colspan="2">0.42</td><td>0.47</td><td>0.2</td><td>0.4</td><td>-</td><td>-</td><td>-</td></tr>
<tr><td rowspan="3">Interphase</td><td>Strong</td><td>0.42</td><td rowspan="3">0.47</td><td rowspan="3">0.2</td><td rowspan="3">0.4</td><td rowspan="3">-</td><td rowspan="3">-</td><td rowspan="3">-</td></tr>
<tr><td>Medium</td><td>0.042</td></tr>
<tr><td>Weak</td><td>0.0042</td></tr>
</table>

Refer to Figure S11. Only a single spherical inclusion (diameter: 0.72 m) was considered in this case to study debonding in the hybrid cube (size: 1 m × 1 m × 1 m). This inclusion was located at the centre of the representative elementary volume (RVE). The inclusion occupied 20% volume of the RVE. The thickness of the interphase was 0.014 m (1/50th of the diameter of the inclusion). A conforming (tetra) type of mesh was used in which the element size and minimum element size parameters were automatically decided based on the volume fraction and number of inclusions which were given as input parameters. Quadratic elements help for a finer control of geometry. Here, we used a much finer mesh for the interphase material by applying body sizing to it. Curvature control helps in adjusting sizes of curved inclusions. Only a single refinement step was taken for each case to avoid too much computational time. Mesh size was taken to be 0.03, for both the matrix and inclusion. With this selected mesh, the RVE was divided into about 50,000 elements in each finite element simulation.

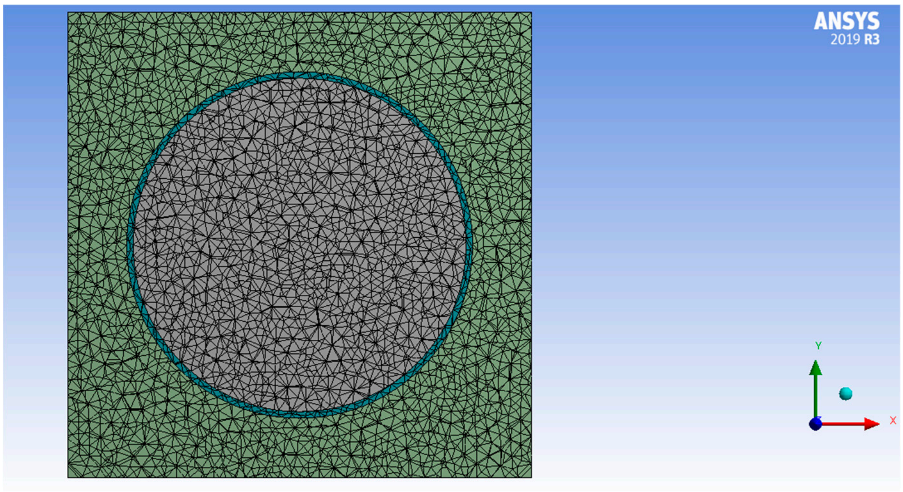

Figure S11. Model and mesh used in simulation.

The simple case of uniaxial loading along the X-axis tension was used in this study. For this particular type of loading, we have used the Dirichlet boundary condition

(displacement type loading), since it saves significantly on the total computational cost and time required to do the analysis.

For finite element analysis, this type of boundary condition was applied using multi-point kinematic constraints between the eight corner nodes of the RVE. These eight corner nodes acted as control nodes, which were acted upon by displacement fields depending on the type of loading, peak strains and loading history. For all nodes lying on each of the six faces of the RVE, the displacement fields were not controlled directly but were interpolated using the multi-point kinematic constraints applied on the eight corner nodes. For this case, the interpolation for a particular face of the RVE was done in a bilinear manner using the four corner nodes of that face. A similar procedure was followed for all six faces. Therefore, the displacement of the RVE was measured relative to these corner nodes.

The loading source used was Digimat, which implied that all loading parameters were user-defined. The loading used was quasi-static loading (strain-controlled), since the stress vs. strain response was independent on the strain rate. The samples in tension were loaded up to 20% strain.

Finally, the file was imported into ANSYS Workbench as a python script and ANSYS Mechanical was used to solve the problem iteratively.

On tensile loading, the RVE started to deform longitudinally and after a particular strain was reached, the interface between the inclusion and matrix began to tear apart or separated along some parts of the contact region. After the maximum strain was reached, an eye-ball type of structure between the centred inclusion and the matrix was observed. Figure S12 indicates the presence of this eye-ball structure formed for all three cases of debonding. Eye-ball structure indicates the creation of microcracks on the edges of the interface between the inclusions and the matrix. It can be clearly seen that for the weak bonding condition, the eye-ball structure is more pronounced than that for other conditions. Hence, it can be inferred that, the weaker the bonding, the higher is the creation of micro-cracks which lead to the formation of micro holes in the interface. As the stiffness of the interface keeps on decreasing, the amount of debonding keeps on increasing and we attain a larger separation in the deformed structure. Figure S12 is for PCL as the inclusion material, which is about 1000 times stiffer than the matrix and hence, it deforms less. In the case of strong bonding, there is a region of high stress present in the central region of the spherical inclusion. Additionally, the strain distributions indicate that since the interface is the least stiff material (its stiffness being less than or equal to that of the matrix), it experiences higher strain regions as seen from the figure using the scale.

The composites were stretched up to 20% strain in the longitudinal direction. The stress values for the case of strong bonding ranged from about 0.07–0.42 MPa, for the case of medium bonding about 0.04–0.4 MPa and for the case of weak bonding about 0.03–0.2 MPa.

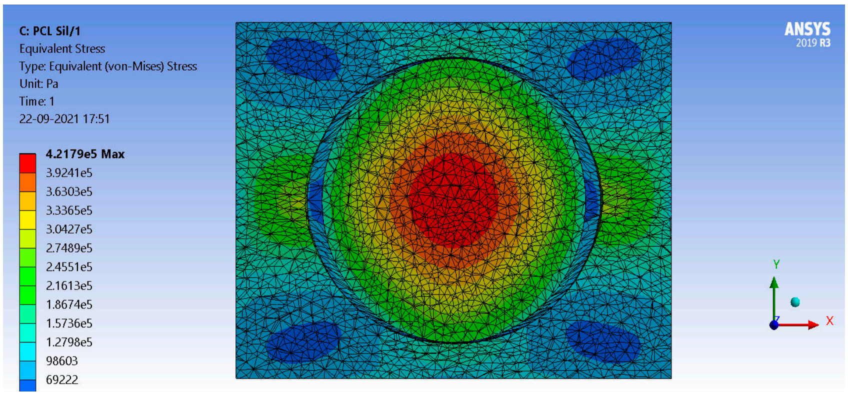

(**a**)

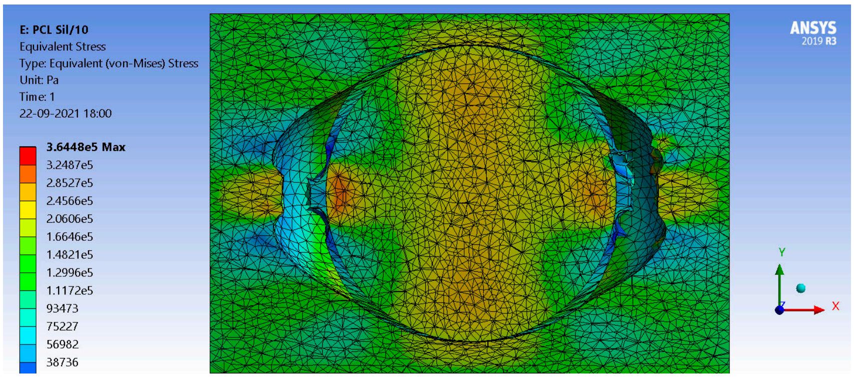

(**b**)

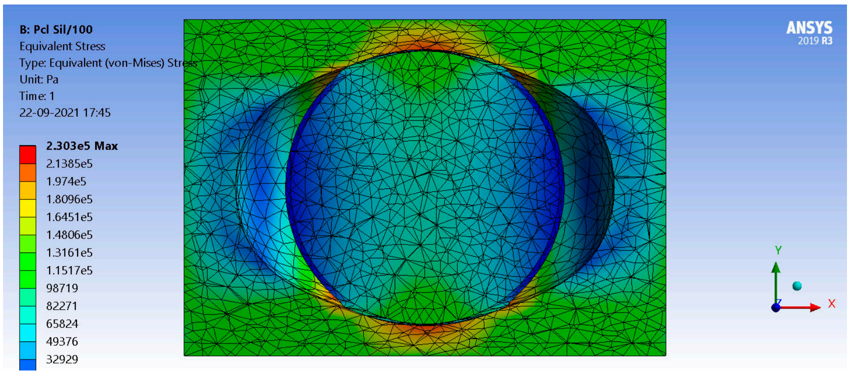

(**c**)

Figure S12. Eye-ball phenomenon (distribution of equivalent stress). (**a**) Strong bonding; (**b**) medium bonding; (**c**) weak bonding.

The corresponding stress vs. strain curves of the samples with the three different bonding conditions (strong, medium and weak) are plotted in Figure S13 for both PCL and PLA inclusions.

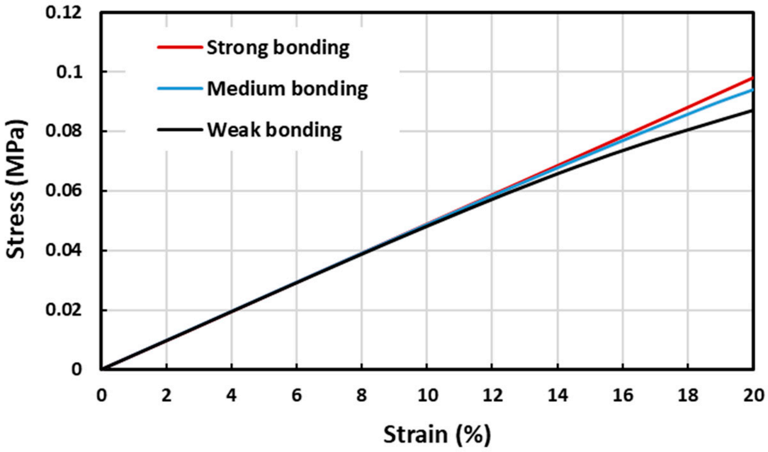

(**a**)

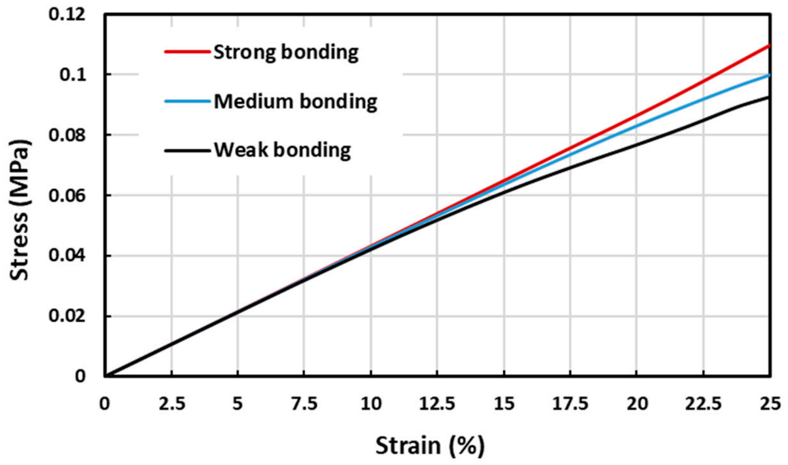

(**b**)

Figure S13. Stress vs. strain response of silicone/PCL hybrids (**a**) and silicone/PLA hybrids (**b**) under tensile loading up to 20% strain for strong (red), medium (blue) and weak (black) bonding.

References

1. Eshraghi, S.; Das, S. Mechanical and Microstructural Properties of Polycaprolactone Scaffolds with One-Dimensional, Two-Dimensional, and Three-Dimensional Orthogonally Oriented Porous Architectures Produced by Selective Laser Sintering. *Acta Biomater*. **2010**, 6, 2467–2476, https://doi.org/10.1016/j.actbio.2010.02.002.
2. Salvekar, A.V.; Zhou, Y.; Huang, W.M.; Wong, Y.S.; Venkatraman, S.S.; Shen, Z.; Zhu, G.; Cui, H.P. Shape/Temperature Memory Phenomena in Un-Crosslinked Poly-ℇ-Caprolactone (PCL). *Eur. Polym. J.* **2015**, 72, 282–295, https://doi.org/10.1016/j.eurpolymj.2015.09.027.
3. Wang, C.C.; Huang, W.M.; Ding, Z.; Zhao, Y.; Purnawali, H.; Zheng, L.; Fan, H.; He, C.B. Rubber-Like Shape Memory Polymeric Materials with Repeatable Thermal-Assisted Healing Function. *Smart Mater. Struct.* **2012**, 21, 115010, https://doi.org/10.1088/0964-1726/21/11/115010.
4. Naveen, B.S.; Naseem, A.B.M.; Ng, C.J.L.; Chan, J.W.; Lee, R.Z.X.; Teo, L.E.T.; Wang, T.; Nripan, M.; Huang, W.M. Body-Temperature Programmable Soft-Shape Memory Hybrid Sponges for Comfort Fitting. Polymers 2021, 13, 3501, https://doi.org/10.3390/polym13203501.
